# Modeling of Arylamide Helix Mimetics in the p53 Peptide Binding Site of hDM2 Suggests Parallel and Anti-Parallel Conformations Are Both Stable

**DOI:** 10.1371/journal.pone.0043253

**Published:** 2012-08-20

**Authors:** Jonathan C. Fuller, Richard M. Jackson, Thomas A. Edwards, Andrew J. Wilson, Michael R. Shirts

**Affiliations:** 1 Astbury Centre for Structural Molecular Biology, University of Leeds, Leeds, United Kingdom; 2 Department of Chemical Engineering, University of Virginia, Charlottesville, Virginia, United States of America; 3 School of Chemistry, University of Leeds, Leeds, United Kingdom; Bioinformatics Institute, Singapore

## Abstract

The design of novel α-helix mimetic inhibitors of protein-protein interactions is of interest to pharmaceuticals and chemical genetics researchers as these inhibitors provide a chemical scaffold presenting side chains in the same geometry as an α-helix. This conformational arrangement allows the design of high affinity inhibitors mimicking known peptide sequences binding specific protein substrates. We show that GAFF and AutoDock potentials do not properly capture the conformational preferences of α-helix mimetics based on arylamide oligomers and identify alternate parameters matching solution NMR data and suitable for molecular dynamics simulation of arylamide compounds. Results from both docking and molecular dynamics simulations are consistent with the arylamides binding in the p53 peptide binding pocket. Simulations of arylamides in the p53 binding pocket of *h*DM2 are consistent with binding, exhibiting similar structural dynamics in the pocket as simulations of known *h*DM2 binders Nutlin-2 and a benzodiazepinedione compound. Arylamide conformations converge towards the same region of the binding pocket on the 20 ns time scale, and most, though not all dihedrals in the binding pocket are well sampled on this timescale. We show that there are two putative classes of binding modes for arylamide compounds supported equally by the modeling evidence. In the first, the arylamide compound lies parallel to the observed p53 helix. In the second class, not previously identified or proposed, the arylamide compound lies anti-parallel to the p53 helix.

## Introduction

### Background

The interaction between the E3 ubiquitin ligase *h*DM2 and a helical peptide forming part of the p53 tumor suppressor domain is of great interest as a target for protein-protein interaction inhibition [1]. Researchers have shown this interaction regulates the tumour suppression function of p53 and thus inhibiting this interaction could be used to treat various types of cancers [1]. Several drugs targeting this interaction are in clinical trials [2]. The system is well studied from a biochemical perspective [3], and, importantly for this study, there is a wealth of structural data giving insight into the mechanism of p53 recognition by *h*DM2[4]–[6].

High quality X-ray structural data for the *h*DM2-p53 system exists. This complex is representative of several helix-mediated protein-protein interactions [7] and has thus served as a popular model system. The *h*DM2 protein structure was first solved in complex with a 15-mer wild-type p53 peptide (SQET**F**SDL**W**KL**L**PEN) by Kussie *et al.* [4]. The peptide was show to bind in an α-helical conformation to a cleft on *h*DM2 with key side chains Phe 19, Trp 23 and Leu 26 from p53 making key contacts with *h*DM2. Grasberger and colleagues determined the structure of a p53 related helix that had been optimized to bind *h*DM2 with higher affinity than the wild-type helix [5]. The nine-mer high affinity peptide (R**F**MDY**W**EG**L**) retains the key binding pattern Phe-Trp-Leu that targets the deep hydrophobic pocket present on the *h*DM2 surface. The wild-type helix is 15 residues long and has a calculated binding affinity (K_d_) of 600 nM [4]. It has been shown that in general shorter helices containing the conserved Phe-Trp-Leu motif will bind more tightly [8].

Small-molecule inhibitors of the *h*DM2-p53 interaction have also been discovered and their atomic structures published [6], [9]. One class of compounds that inhibits *h*DM2 by targeting the p53 pocket is the Nutlins. In 2004, Fry *et al.* published the NMR structure of *Xenopus Laevis h*DM2 bound to a small-molecule inhibitor [9] belonging to this Nutlin family described in the work by Vassilev *et al*. [6]. Vassilev *et al.* published the structure of *h*DM2 in complex with a *cis-*imidazoline compound to 2.3 Å resolution [6]. The authors screened a diverse range of compounds identifying the *cis-*imidazoline compounds as promising lead compounds. One of these compounds, Nutlin-2, was measured to have an IC_50_ of 0.14 µM. An improved IC_50_ of 0.09 µM was determined from an enantiomer of a related compound called Nutlin-3a, and specificity was demonstrated via the 200-fold lower affinity of the enantiomer Nutlin-3b [6].

Helix-mediated PPIs such as p53-*h*DM2 represent a class of interaction within the wider family of PPIs that may be amenable to the elaboration of general approaches for small molecule modulation [7]. Hence, the structure of the *h*DM2 binding pocket has been widely investigated both experimentally and computationally because it has features that suggest it is a ‘druggable’ protein-protein interface [10]. NMR studies of the *h*DM2 binding pocket show the unbound structure to be flexible [11]. McInnes *et al.* specifically note that on binding there is rearrangement of residues in the p53 binding site compared to the NMR structure of the apo protein. The flexibility of the binding site brings into question whether it is possible to use structure-based methods to design protein-protein interaction inhibitors given only the apo structure of protein binding partners. Eyrisch and Helms previously investigated three protein-protein interactions including the *h*DM2-p53 interaction using molecular dynamics simulations and the PASS algorithm for pocket detection [12]. They concluded that ligand binding pockets are observed opening and closing over tens of picoseconds in their nanosecond simulations [12]. Carotti *et al.* studied the *h*DM2 and related *h*DMX system computationally to attempt to identify key residues involved in p53 binding [13]. They used 60 ns MD simulations to investigate structural changes between the bound and unbound forms of each protein. In these simulations they identified that a change in conformation of Tyr 99 from *h*DM2 results in a decrease in *h*DM2 solvent accessible surface area (SASA) when moving from the apo to bound states [13].

There have also been many experimental efforts to design inhibitors of the *h*DM2-p53 interaction. Experimental high throughput screening (HTS) methods in combination with use of crystal structures identified the initial inhibitors of the *h*DM2 interaction. Wang and co-workers used the GOLD docking program to identify spiro-oxyindole-based scaffolds that could inhibit the *h*DM2-p53 interaction with micromolar affinities [14], successfully identifying several novel inhibitors of *h*DM2. Peptide libraries developed using phage display technologies have been used to find novel peptide binders for *h*DM2 [15]. This technique has many advantages due to its ability to screen large libraries of peptides with similarities to the p53 peptide relatively inexpensively [15].

Several computational studies attempting to identify key binding site features and compute ligand binding free energies have been performed previously, although often over timescales of less than 5 ns for individual trajectories[16]–[19]. Massova and Kollman studied the *h*DM2-p53 interaction using a technique that they developed named computational alanine scanning [16]. They used an MM-PBSA model to estimate the free energy change required to mutate a side-chain to alanine. This allowed them to propose key residues contributing to the binding energy and identified a direct correlation between the energy and the frequency of accepted mutations in homologous p53 peptides. Similar work was performed by Kortemme and Baker, using a simple model using empirical statistical potentials [17]. They identified residues on both the p53 peptide and the *h*DM2 binding site that contribute significantly to binding free energy with results correlating with the free energies estimated by Massova and Kollman. These studies suggest that simulations of *h*DM2 can provide useful information on the *h*DM2-p53 interaction.

Novel classes of inhibitors of protein-protein interactions, such as the *h*DM2-p53 interaction, are of great interest due to the involvement of such interactions in a number of disease pathways. A variety of generic templates have been described as inhibitors of such helix mediated PPIs [7], including constrained peptides [20], β [21], or α/β-peptides [22] and proteomietics [23]. Proteomimetics are non-natural scaffolds that present functional groups in a similar 3D arrangement to natural protein side chains; such scaffolds can in theory be used to modulate any protein-protein interaction of interest. In this study, we computationally investigate the binding of arylamides, foldamer compounds designed to mimic the p53 transactivation domain α-helix and competitively inhibit the *h*DM2-p53 interaction [24], [25]. 3-alkoxysubstituted-*para*-oligoarylamides adopt a rod-like conformation that can present side-chains at locations similar to those at the i, i+4, i+7 locations on an α-helix [26]. These side-chain locations correspond to the Phe-Trp-Leu residues identified as hot-spot residues for the *h*DM2-p53 interaction. The compounds investigated in this study are synthetically accessible using an iterative approach that sequentially couples then reduces 4-nitro-3-alkoxybenzoic acid monomers. [27] A representative X-ray structure of these arylamide compounds revealed an intramolecular hydrogen bond between the amide NH and ether oxygen [27]. This finding is mirrored in results from solution NMR of the arylamide compound in deuterated DMSO-d_6_ and CDCl_3_ [27]. 2D ^1^H–^1^H NOESY spectra, indicating that there is free rotation about the ArCO bond. This intramolecular hydrogen bonding restricts rotation about the ArNH bond [27]. Model arylamide dimers show similar configurational preferences [28].

Further work by Plante *et al.* showed that arylamide compounds can act as low µM inhibitors of the *h*DM2-p53 interaction [26]. Shaginian *et al.* described the synthesis of a library containing a diverse series of these arylamide compounds [29]. A limited docking study was performed by Shaginian *et al.* identifying configurations presenting the arylamide side chains coincident with the locations of the p53 Phe-Trp-Leu side chains. In the work by Plante *et al.* six arylamides were synthesized and screened against the *h*DM2-p53 interaction using a fluorescence anisotropy assay, showing that the compounds inhibited the *h*DM2-p53 interaction with IC_50_’s of between 10 µM and 1 µM [26]. However, there are no published structures of *h*DM2 in complex with an arylamide compound, which motivated this study to identify putative models of the complex.

Computational studies have also helped us understand the properties of arylamide compounds. A quantum mechanics study of the torsional profile of arylamide compounds calculated the location of minima and the heights of barriers between minima for the C_a_-C_a_-C-N, C_a_-C_a_-N-C dihedral angles, data required to identify force field parameters that can accurately model the arylamide [30]. Molecular dynamics has been used to investigate the behavior of arylamide compounds designed to mimic heparin in solution. For example, Pophristic *et al.* identified problems with adequately sampling certain arylamide backbone conformations using standard molecular dynamics approaches and used an enhanced sampling technique to attempt to overcome the problems [31]. Vemparala and co-workers noted that altering the thioether to an ether group is one way in which the flexibility of the compound could be controlled, since the larger thioether group would reduce backbone flexibility [30].

### Work Carried Out in this Study

As there are no published structures of any *h*DM2-arylamide complexes, computational insights into this complex can significantly aid design of improved arylamide inhibitors. In this study we use molecular docking to generate conformations of arylamide compounds bound to *h*DM2. We identify appropriate parameters that result in experimentally observed arylamide backbone torsional preferences. We then analyze these conformations and use the best ranked ones to perform molecular dynamics calculations of the bound systems. We use these molecular dynamics simulations to test whether *h*DM2-arylamide complexes behave similarly to p53-*h*DM2 complexes or other small-molecule inhibitor complexes. We then analyze dynamics of the arylamide dihedral angles and spatial sampling in the *h*DM2 binding pocket to identify problems in the configurations found by molecular docking. These molecular dynamics simulations also allow us to identify how long simulations of *h*DM2 bound to arylamides may need in order to reach convergence, and whether a single bound starting conformation is sufficient to calculate equilibrium thermodynamic properties.

## Methods

### Structural Superposition

We performed structural superposition of proteins with UCSF Chimera version 1.4 on the Mac OS X operating system using the MatchMaker function with default settings [32], in order to compare binding modes between peptides and small-molecule ligands. The *h*DM2 chains (1Z1M-model 9, 1YCR-chain A, 1T4F-chain M, 1RV1-chain A, 1T4E-chain B) were superposed using the MatchMaker algorithm, while the bound ligands–where present–were subjected to the same rotation and translation as their partner proteins. The ligand is therefore retained in the same position relative to partner protein, and all ligands can be compared in their common binding site.

### Docking

There are no known structures for *h*DM2 bound to arylamide compounds. However, computational docking of these compounds to *h*DM2 generates physically reasonable complexes that can be further evaluated using molecular dynamics. Two rounds of docking using Autodock were performed [33], [34]. The first round was used to assess the performance of the Autodock force field, whereas the second round was used to generate plausible conformations of the *h*DM2-arylamide interaction. In both docking rounds, random number seeds were generated from the Autodock PID and the current system time. The protein structure used was derived from the structure of *h*DM2 bound to a high-affinity p53 peptide (1T4F-chain M), with all water molecules and the helix removed, protonation states manually assigned taking into account the local hydrogen bonding network in the vicinity of histidines which can have multiple protonation states at pH 7. A grid centered on 13.119, 18.969, 10.941 was used with spacing of 0.375 Å and 52, 58 and 48 points in the x, y and z directions.

In the first round of docking, we used Autodock 4.0 to perform 2.5 million evaluations for 27,000 generations with population size 300 to produce 101 docked conformations. The results from this set of dockings were clustered at a 2 Å RMS cutoff. The lowest energy representative structures of these clusters were used in the initial MD simulations and are representatives from the largest low energy clusters labeled clu1, clu2, clu3 (Figure S1 in Text S1). The second round of docking calculations were performed with Autodock 4.2.1 using a Lamarkian genetic algorithm. 150 docked conformations were generated, with each using 25 million evaluations for 27,000 generations of population size 300. We also used the docking program FRED (OpenEye, version 2.2.5) to see if the results were independent of docking program. FRED is a rigid body docking program, meaning that conformations of the molecule to be docked are generated prior to docking. Conformations for the arylamide compounds were generated using the OMEGA (OpenEye, version 2.2.1) conformational generator, supplied by OpenEye. OMEGA uses an energy window of 25 to generate a maximum of 1 million conformers (maxconfgen), of which a maximum of 10000 with RMSD of greater than 0.5 Å between previously generated conformers were kept (maxconfs). 150 docked poses were generated, with all settings not mentioned left as default.

### Preparation of Structures for Molecular Dynamics

All structures were taken from the Protein Data Bank (PDB) [35]. In cases where multiple chains were present, a single *h*DM2 chain was selected: 1T4E-A; 1T4F-M; 1YCR-A; 1RV1-A; 1Z1M model 9. We selected the corresponding bound ligand where appropriate: 1T4E-A; 1T4F-P; 1YCR-B; 1RV1-A. All water molecules were removed from the crystal structures and protonation states were manually assigned using the same criteria as described in the section ‘Docking’. Ligand molecules were parameterized with GAFF parameters and AM1BCC charges using the default settings from the acpype front end to Antechamber [36]–[38]. The grompp program from Gromacs was used to assign AMBER99sb force field parameters from the ffamber ports [39]–[42].

GAFF parameters for the ArCO and ArNH dihedral angles in the arylamide compounds were replaced by those reported by Vemparala *et al.* [30], since we were predominantly interested in the correct location of minima in the torsions. Work by Liu *et al.* (published after the computations in this study were performed) validates the choice of these modified parameters for the ArNH bond (–SCH_3_ containing model arylamide compounds) for the ArNH dihedral for the arylamide compound in this study (–OCH_3_ functional group) [43]. Liu *et al.* showed that the potential energy profile of the ArNH dihedral follows the same dihedral pattern for model compounds containing the –OCH_3_ and –SCH_3_ functional groups bonded to the benzamide ring, with barrier heights within 1 kcal mol^−1^ for both compounds [43].

### MD Simulations of p53 and Small-molecule Inhibitors of HDM2

In order to determine whether our molecular dynamics protocol is appropriate for simulating the *h*DM2 system, including the specific force field choice of AMBER99sb/GAFF, we first performed simulations of *h*DM2 inhibitors of known structures. Initial MD simulations were performed using Gromacs 3.3.1 [41]. All structures were minimized to a tolerance of 100 kJ mol^−1^ nm^−1^ with an initial step size of 0.01 nm for a maximum of 5000 steps of L-BFGS minimization with 10 correction steps, followed by a maximum of 500 steps of steepest descent minimization due to occasional early terminations of the Gromacs implementation of L-BFGS. Minimization was followed by 10 ps of isothermal dynamics followed by 100 ps of isothermal/isobaric equilibration using the Berendsen algorithms [44]. Production simulations were run for a total of 10 ns. In the latter two stages pressure coupling was performed using a Berendsen barostat with reference pressure of 1 atm, compressibility of 4.5×10^−5^ bar^−1^ and relaxation time of 0.5 ps. All simulations used the Gromacs stochastic integrator (sd) with reference temperature 300 K and relaxation time 0.1 ps for the entire system, with a step size of 2 fs. PME parameters are from Mobley *et al.* [45], with PME spline order of 6, relative tolerance of 1×10^−6^ and a Fourier spacing of 0.1 nm. A long-range dispersion correction is also applied for energy and pressure to correct for the truncation of the long-range dispersive interactions. A Lennard-Jones function with switching between 0.8 nm and 0.9 nm was used for the van der Waals interactions. The neighbor list was set to 1 nm and updated every 10 simulation steps. All bonds with H-atoms were constrained using the LINCS algorithm with highest order expansion of the constraint coupling matrix of 12. SETTLE was used to constrain water bonds and angles.

### Arylamide MD Simulations

We used molecular dynamics simulations to investigate the dynamics of the *h*DM2 binding site and compounds with known structures of *h*DM2 complexes. Molecular dynamics simulations were performed using Gromacs, version 4.0.4 [42]. Simulations were performed using conformations generated from the second round of Autodock docking (5 anti-parallel and 4 parallel conformers labeled conf. 1, 2, 3, 7, 8 and conf. 4, 9, 10, 11 respectively). All docked structures were minimized to a tolerance of 100 kJ mol^−1^ nm^−1^ with an initial step size of 0.01 nm for a maximum of 5000 steps of L-BFGS minimization with 10 correction steps, followed by a maximum of 2000 steps of steepest descent minimization. Minimization was followed by 10 ps of isothermal dynamics followed by 100 ps of isothermal/isobaric equilibration using the Berendsen algorithms. Production simulations were run for 20 ns each. In the latter two stages pressure coupling was performed using a Parrinello-Rahman barostat with reference pressure of 1 atm, compressibility of 4.5×10^−5^ bar^−1^ and relaxation time of 5.0 ps.

As previously described for the MD simulations of p53 and small-molecule inhibitors of hDM2, data for the *h*DM2/arylamide complexes was generated using simulations using the stochastic integrator with reference temperature 300 K and temperature relaxation time 0.1 ps for the entire system, with a step size of 2 fs. All PME, long-range dispersion, cutoff, neighbor list, and constraint parameters were the same as with other Gromacs simulations in this study.

### Analysis of Gromacs Simulations

Molecular dynamics simulations were first analyzed to check for convergence in several standard properties such as temperature and energy. Gromacs simulations were then analyzed using four key measures: the RMSD from the initial structure (after two rounds of minimization) throughout the time-course of the simulation (using the g_rms tool); the RMSF of individual residue Cα atoms from the initial structure after two rounds of minimization (using the g_rmsf tool); the number of intermolecular pairs of atoms between *h*DM2 and the ligand that are with 3.5 Å (using the g_hbond tool); and the difference in the distance between the center of mass of the *h*DM2 molecule and the bound ligand molecule compared to the initial structure after two rounds of minimization (using the g_dist tool).

### Dihedral Analysis

Dihedral angles were monitored during the simulations, as it is known that dihedrals are often not well sampled even in extremely long time-scale simulations of protein-ligand binding sites [46]. The distribution of dihedral angles over 20 ns of production simulation is plotted for each χ angle for all residues contacting the ligand and each dihedral present in the arylamide. Additionally, the starting value of each dihedral is marked on the distribution. We analyzed the distributions, identifying dihedral angles sampled in many simulations but missing in other simulations. Angles are labeled as: ‘well sampled’ where all simulations sample the same distribution; ‘mostly well sampled’ where all but one simulation samples the same distribution, or some peaks are considerably different in height but still sampled and located at the same angle; and ‘possible sampling problem’ where peaks are missing from more than one simulation indicating that some starting conformations can access dihedral angles that others may not be able to access.

### Autocorrelation Analysis

We computed the autocorrelation function of the cosine of the dihedral angles to compare the timescale of these dihedral rotations to the timescale of our simulations. Autocorrelation functions of length 10 ns (from simulations of length 20 ns) were generated for each χ angle from *h*DM2 binding residues for 5 anti-parallel and 4 parallel starting conformations of the Phe-Nap-Leu compound. The autocorrelation function was fit to an exponential of the form y =  exp(−x/τ) using the g_chi program from Gromacs 4.0.4 [42]. Numerical integration of the exponential, also carried out using g_chi, yields the relaxation time for the χ angle.

### Orientation and Positioning of Arylamide Compounds in the HDM2 Binding Pocket

Investigating the orientation of the arylamide compounds relative to the *h*DM2 binding pocket allows us to ask whether the ligands in all simulations tend to converge to the same region of space in the pocket. This would indicate strongly that there is a clear preferred binding mode and additionally mean that the choice of starting configuration for simulations is less important. Spatial sampling was analyzed by projecting the position of each of the three ether oxygen atoms of the arylamide from 20 ns simulations at time intervals of 10 ps onto a plane defined by the Cα atoms of Tyrosine 56, Methionine 62 and Valine 93. These three atoms lie in the periphery of the binding site and define a plane that cuts through the site at a roughly constant depth. A Python program using the Numpy toolkit was written solving the equation describing the intersection of a line *l*, and a plane *p*:
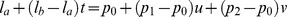



This algorithm calculates the projection along the direction normal to the ligand plane containing the point (l_b_) and an ether oxygen atom at position l_a_ (defining a line (l_b_–l_a_)*t*) onto the plane defined by the Cα atoms of the protein (p) at p_0_ (point defined by Cα Tyr 56), the line defined by (p_1_–p_0_)*u* (where p_1_ is the point defined by Cα Met 62), and the line defined by (p_2_–p_0_)*v* (where p_2_ is the point defined by Cα Val 93).

### Cluster Analysis of Arylamide Conformations

The Gromacs 4.0.4 program g_cluster was used to generate clusters with a minimum RMSD of 1.5 Å. The clustering method takes a random structure from the pool of structures and identifies all structures within the RMSD threshold, defining a cluster. The structure with the most neighbors from the largest cluster is selected as the group center, and this structure and all of its cluster members are removed from the pool. The procedure is repeated until the pool of structures is empty and all structures are assigned to clusters [47]. Cluster size (number of members of each cluster), and cluster membership was generated for the pooled conformations taken at 10 ps intervals between 3 ns and 20 ns from 5 anti-parallel (1, 2, 3, 7, 8) starting conformations. 3 ns is chosen as the point where temperature, pressure and other short timescale fluctuations had equilibrated. The same was repeated for the 4 parallel (4, 9, 10, 11) starting conformations.

## Results and Discussion

We first address the suitability of the MMFF94, Autodock and GAFF force fields for modeling arylamide compounds and using molecular docking to generate *h*DM2-arylamide complexes. We then describe the results of molecular dynamics simulations on these putative complexes to help validate the bound configurations of arylamide compounds in the *h*DM2 binding pocket and provide an insight into arylamide binding. We assess the sampling quality of *h*DM2 arylamide interactions examining side chain dihedrals of binding site residues and of arylamide compounds, arylamide conformational clustering, and orientation and positioning of arylamide compounds in the *h*DM2 binding pocket. Besides providing understanding of the structural interactions of these ligands, these simulations also highlight the importance of proper sampling in both docking and molecular dynamics with respect to previously performed studies.

### Force Fields Describing the Behavior of Arylamide Compounds

We observed that conformers generated by OMEGA described the non-standard behavior of the ArCO and ArNH bonds when using the MMFF94 force field, reported experimentally by Plante *et al.* and Prabhakaran *et al.*, and *in-silico* by Vemparala *et al*. [27], [28], [30]. While AutodockTools correctly identified the amide bond present in the arylamide compound as rigid, it could not describe the planar conformation of the ArNH torsion and the free rotation about the ArCO bond (Figure S2 in Text S1). The position of the ArNH torsion in Figure S2 lies at the peak of a metastable region identified by Vemparala *et al.* Furthermore, QM calculations showed that the torsion angle is about 6 kcal/mol greater in energy than its most stable energy minimum, thus an unlikely conformation [30]. Using the parameters from the thioether compound previously studied by Vemparala *et al.* the ArNH and ArCO torsion conformational preferences are described much more accurately. Indeed, the use of these thioether parameters in place of ether parameters has been validated by a study published since the simulations that we report were carried out [43]. As a result, all further Autodock computations restrained the arylamide with the ArNH dihedral oriented so that the amide hydrogen can form the intramolecular hydrogen bonds with the ether oxygen that are observed in X-ray structure and NMR data of the uncomplexed ligand [27], [28].

### Molecular Dynamics Simulation of an Arylamide Compound in Solution

**Figure 1:**
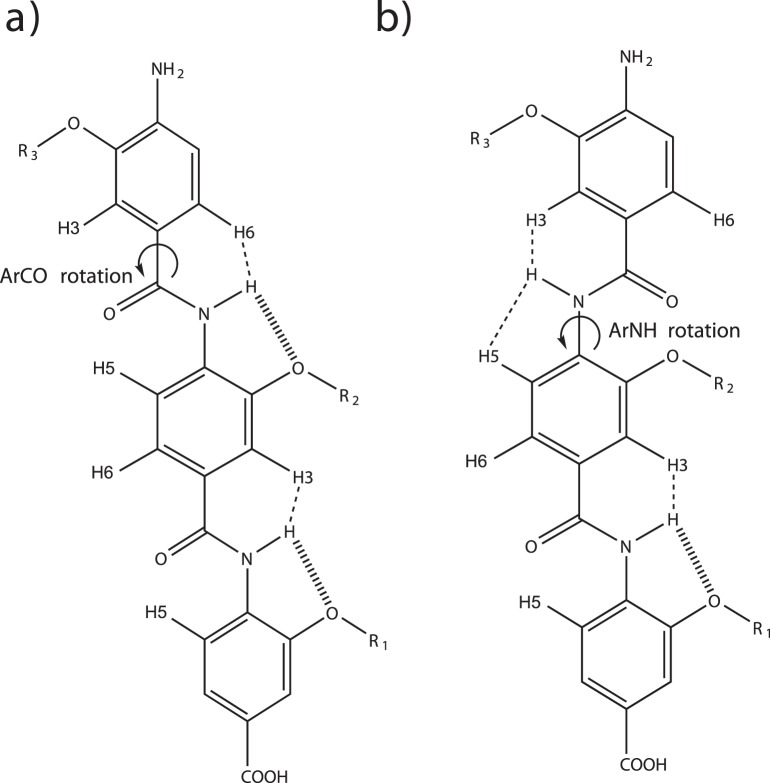
2D Structure of the arylamide backbone. a) hydrogen bonding pattern with free rotation of the third oligomer around the ArCO bond as shown by the arrow. In this case the amide H–H3 and the amide H–H6 can both be in close proximity, but the H–H5 protons can not. This pattern was observed in NMR experiments and dihedral-modified GAFF parameter MD simulations. In b), no hydrogen bond forms leading to free rotation of the third residue around the ArNH bond. In this case the amide H–H3 and the amide H–H5 protons can both be in close proximity, but the amide H–H6 protons can not. This pattern was observed in the unmodified GAFF parameter MD simulations of arylamides, but not in NMR experiments.

Comparing molecular dynamics simulations of an arylamide compound in solution with and without the dihedral-modified GAFF parameters show that our modified GAFF parameters describe the experimentally observed free rotation about the arylamide ArCO bond, whereas the unmodified GAFF parameters do not show free rotation about the ArCO bond. Prabhakaran *et al.* published 2D ^1^H–^1^H NOESY NMR spectra of 2-*O*-alkylated arylamide model compounds, with napthyl, isobutyl or benzyl side groups [28]. From the presence of NOEs between the NH and aromatic protons on the adjacent ring they were able to observe free rotation about the ArCO bond. However, the lack of an NOE from the NH to the anilide ring confirms that the ArNH torsion is restrained by hydrogen bonding to the ether oxygen. We used a 1/r^3^ distance average from our 20 ns trajectories to allow qualitative comparison to the 2D ^1^H–^1^H NOESY NMR spectra presented by Prabhakaran *et al*. and we use the same nomenclature for the H2, H5 and H6 protons (see Figure 1) [28]. Using the unmodified GAFF force field the distances between the amide proton and the adjacent aromatic benzamide protons were 1.8 Å and 4.4 Å respectively, so one would expect to observe an H2 NOE but not an H6 NOE. The absence of an H6 NOE is in contradiction to the NMR data, and shows that free rotation around the ArCO bond is not possible when using the unmodified GAFF parameters. The amide proton to benzamide H5 proton distance is 3.3 Å which shows that the ArNH bond has restricted ability to rotate when using the unmodified GAFF parameters. For MD simulations using the modified GAFF parameters, comparing the 1/r^3^ average distances observed for protons from the arylamide scaffold containing the carboxy-terminal Leu mimic, and the central Trp mimic, we observed that the distance between the amide proton and the H2 and H6 benzamide protons was 2.5 Å and 2.4 Å respectively. Since the simulations show that both H2 and H6 distances are equivalently small one would expect to observe both H2 and H6 NOEs. The presence of both H2 and H6 NOEs is indeed in agreement with NMR data and indicates that free rotation around the ArCO bond is possible when using the modified GAFF parameters. The MD simulations using modified GAFF parameters show that the 1/r^3^ average distance between the amide proton and the adjacent H5 benzamide proton is 3.3 Å which shows that the ArNH bond has restricted ability to rotate as is observed in the NMR experiments of Prabhakaran *et al*. [28].

### Structural Superposition Shows that Current Small-molecule Inhibitors Bind in a Manner Similar to the p53 Peptide

Key to any study of the *h*DM2-arylamide protein-ligand complex is an accurate structure for the protein-ligand complex. Ideally this would come from X-ray or NMR structures; however, no such structures exist for arylamides. However, as discussed earlier in the case of *h*DM2, there is an NMR structure of the free protein in addition to high-resolution X-ray structures of the protein bound to a wild-type p53 helix, a high-affinity p53 helix, a benzodiazepinedione compound and Nutlin-2. The former two are peptides while the latter two are small-molecules specifically designed to target this interaction.

**Figure 2:**
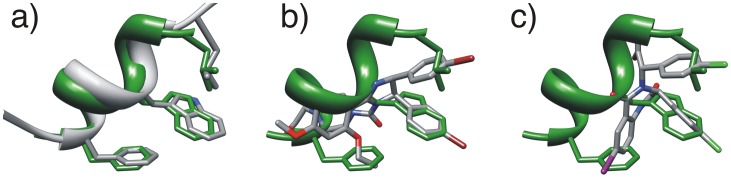
Superposition of the ligand bound *h*DM2 structures. Benzodiazepinedione and Nutlin-2 compounds both target the same regions of space corresponding to the Phe-Trp-Leu motif from p53. Representations of high affinity helix (green) shown relative to: a) wild type helix; b) Nutlin-2; c) Benzodiazepinedione compound. Figures were generated using the matchmaker function from Chimera to superpose hDM2 from PDB code 1T4F to pdb codes: a) 1YCR; b) 1RV1; c) 1T4E.

Structural superposition of different *h*DM2 protein-ligand complexes reveals that the two reported inhibitors benzodiazepinedione and Nutlin-2 target the same regions of the binding pocket as the high-affinity p53 peptide, mimicking the interaction of Phe-Trp-Leu side-chains from the p53 peptide as seen in Figure 2. Both series of inhibitors were discovered through independent high-throughput screens. They both have scaffolds that allow the presentation of their key functional groups in very similar spatial locations to the high-affinity peptide, indicating that use of a common backbone to design inhibitors is likely a useful strategy.

In Figure 2b, we can see that the Nutlin-2 compound closely mimics the binding mode of the high-affinity p53 helix. The two chlorophenyl groups target the Leu and Trp pockets and the arylethyl ether moiety binds in the Phe pocket. The crystal structure of *h*DM2 bound to a high-affinity helix was reported at the same time as a 2.6 Å structure of *h*DM2 bound to a benzodiazepinedione compound. The benzodiazepinedione compound also mimics the same Phe-Trp-Leu binding mode as the p53 peptides as shown in Figure 2c. Grasberger *et al.* noted that the inhibitor interacts with the *h*DM2 binding pocket through non-specific van der Waals contacts. Because of these patterns, when targeting the *h*DM2 binding pocket with arylamide based helix mimetics we expect that high-affinity compounds should also target these same structural features [5].

### Molecular Docking Supports the Putative Binding Location

**Figure 3:**
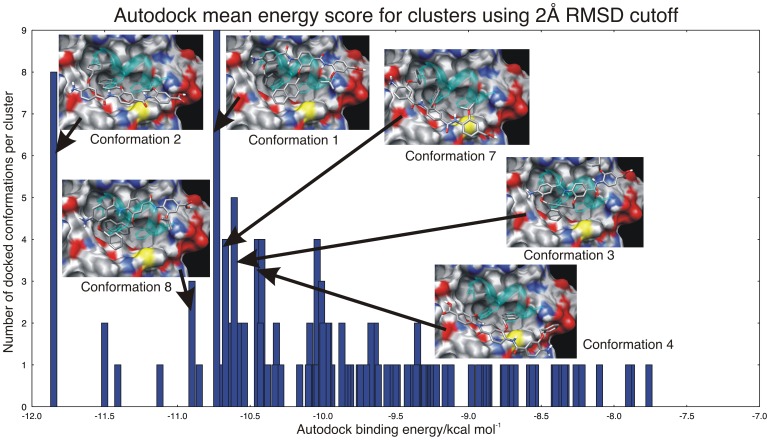
Mean Autodock binding energy score and corresponding cluster occupancy with a 2 Å RMSD cutoff. Representative conformations used as initial conformations for MD simulations and the cluster from which they originated are highlighted. Highly populated clusters with low mutual RMSD all exist within 2.5 kcal/mol, meaning that the docking scoring function is unable to determine a consensus bound conformation.

Due to the lack of experimental data on the structure of the *h*DM2-arylamide interaction we have used two molecular docking programs in addition to using an alternative superposition based method (Figure S3 in Text S1). Our key assumption, based on the evidence presented in the section ‘Structural superposition shows that current small-molecule inhibitors bind in a manner similar to the p53 peptide’, in creating our docking model is that since the four compounds for which we have high resolution structures available all bind to the same site, this site is where the arylamide compounds are most likely to bind. These compounds also experimentally displace the helix from *h*DM2 [23], [26]. Furthermore, since the arylamide compounds have been designed to mimic the side-chains present at positions i, i+4, i+7 on an α-helix, we expect to find arylamide substituents bound at these sites. This is supported by structural superposition of the *h*DM2 protein from the putative docked arylamide structures to the *h*DM2 protein from the p53 peptide bound structure, allowing comparison of the relative location of the arylamide compound and the p53 peptide (Figure S1 in Text S1). Since there are no structures of immediately similar compounds to the arylamides in which we were interested, and there is no data about binding affinities for many of these compounds, we were limited in our ability to assess the quality of the results produced by docking programs. We generated 150 docked poses, with the ArNH dihedral restrained to preferred low energy conformations identified by OMEGA, to select a small number of compounds for use in MD simulations of the *h*DM2 binding site. We identified three possible docking modes from the top three low energy clusters using a 2 Å RMS clustering threshold. The resulting representatives from each cluster are shown in Figure S1 (Text S1) relative to the position of high affinity p53 helix. These cluster representatives were produced by identifying the rotation and translation that maps the *h*DM2 atoms used in the docking run onto the 1T4F atoms, and applying the same rotation and translation to the arylamide compound, allowing comparison of the docked compounds to that of the high-affinity p53 peptide. This produces two classes of potential binding complexes. The first class consists of parallel conformations, which present their C-terminus spatially proximal to the location of the C-terminus of the p53 helix and their N-terminus spatially proximal to the N-terminus of the helix, such as in conformation 4 in Figure 3. The second class of anti-parallel conformations present their C-terminus spatially proximal to the N-terminus of the p53 helix and their N-terminus spatially proximal to the C-terminus of the p53 helix, such as in conformations 1, 2, 3 and 8 in Figure 3.

### Molecular Docking is Suggestive of a Preference for Anti-parallel Arylamide Conformations

Autodock sampling was performed again with more computationally expensive enhanced parameters that performed ten times more energy evaluations in order to determine whether parallel or anti-parallel *h*DM2-arylamide conformations might be more likely. The results from the enhanced docking simulation are presented in Figure 3, where the mean Autodock binding energy score is presented for each of the clusters generated using a 2 Å RMSD cutoff. The enhanced parameters for Autodock may show some preference for anti-parallel arylamide conformations with a slight bias to anti-parallel modes; however these calculations are only suggestive of a bias towards anti-parallel conformations since docking calculations do not properly sample the full thermodynamic weights of configurations. Representative structures from each of the large low energy clusters are shown inset, alongside a representation of the high-affinity p53 helix shown in cyan (Figure 3). Here we see that conformation 2 has the lowest energy of about −11.8 kcal mol^−1^, while conformation 1 also has a highly populated cluster with mean Autodock energy of −10.8 kcal mol^−1^, a difference of only 1 kcal mol^–1^. We compare the energy of the clusters to identify the likely binding mode, noting that that highly populated clusters within 2.5 kcal mol^-1^ of each other are unlikely to be distinguished from an incorrect binding mode [34]. There is significant literature suggesting that docking experiments are not suitable to predict the binding affinity of protein-ligand complexes, but they can still provide important information [48]. For example, Warren *et al.* found that docking programs often identified the structure of the crystallographic ligand. However, the scoring functions were often not able to identify the structure of the crystallographic ligand as the lowest energy pose [48].

Previous work by Shaginian *et al.* presented only a parallel binding mode when they used Autodock 3. However, because their full methods were not presented, it is impossible to make direct comparison to our results that show both parallel and anti-parallel conformations [29]. The results shown in Figure 3 suggest a possible bias towards anti-parallel conformations, with five of the six large low energy clusters having this orientation. This bias may be due to the fact that arylamide conformers are more stable in their anti-parallel binding orientation, perhaps due to steric clashes. Another hypothesis is that the negatively charged C-terminus of the arylamide is favored in the region of the N-terminus of the p53 helix, since the surface potential is slightly positive in this region (Figure S4 in Text S1).

We also compared results from the rigid-body docking program FRED, which uses conformers of ligands generated by OMEGA and a static representation of the protein molecule, to the results from Autodock. We tested the ability of Chemgauss 3, the standard scoring function included with FRED, to identify likely docked structures. However, we found that with Chemgauss 3 docked arylamides were often very exposed to the solvent in many of the high ranked complexes (Figure S5 in Text S1). This result is unphysical due to the hydrophobic nature of arylamide compounds. When using FRED we observed 49 conformations in the parallel conformation and 101 in the anti-parallel conformation. As with the results from Autodock, even considering potential issues with the Chemgauss 3 scoring function in this complex, these results illustrate a significantly more complex picture than the previously reported docked structure presented by Shaginian *et al.* [29].

### Stable Molecular Dynamics Simulations of HDM2-arylamide Conformations Support the Binding Sites Identified by Molecular Docking

**Figure 4:**
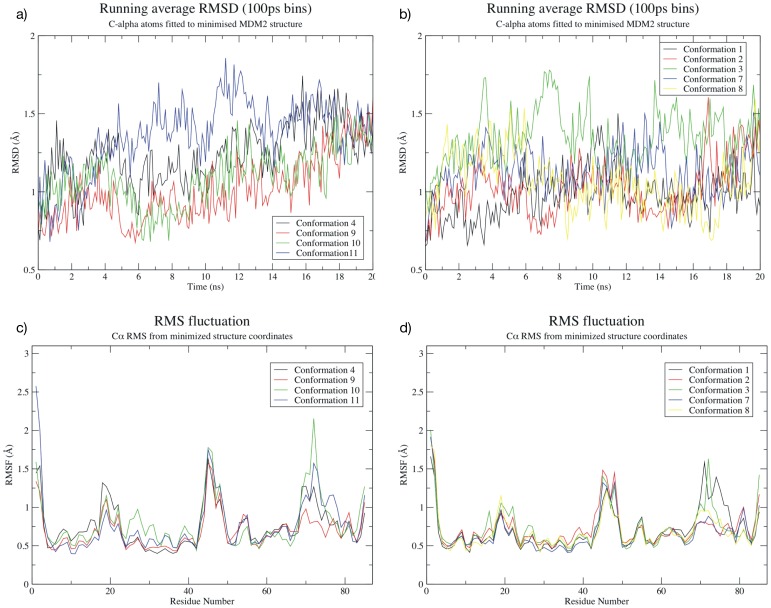
Behavior of parallel (left) and anti-parallel (right) Phe-Nap-^i^Pr conformations of the arylamide compound. a) RMSD relative to initial minimized parallel conformation; b) RMSD relative to initial minimized anti-parallel conformation; c) RMS fluctuation of C-alpha atoms from initial minimized parallel conformation; and d) RMS fluctuation of C-alpha atoms from initial minimized anti-parallel conformation. The RMSD calculations tend to increase over time, but converge towards similar values after longer simulation times. The RMSF calculations exhibit similar behavior to that observed in Figure S6 (Text S1), indicating that similar regions of the protein remain more flexible.

If simulations of the *h*DM2-arylamide system had similar stability to simulations of *h*DM2 complexes with known bound structure, it would suggest that arylamides do indeed bind in the p53 binding pocket as hypothesized by our structural superposition and molecular docking studies. We used a selection of starting conformations generated in the docking results to start our MD simulations. Figure 4 shows the RMSD/RMSF observed in the molecular dynamics simulations. The RMSD from starting structures stays within reasonable limits (<2 Å) and RMSF between replicate simulations of the same complex is comparable. These structural fluctuations are in good agreement with the simulations of *h*DM2-p53, *h*DM2-Nutlin-2 and *h*DM2-benzodiazepinedione which remain similarly stable with equivalent RMSF (Text S1, Figure S6 and Figure S7). Specifically, both parallel and anti-parallel arylamide starting conformations have an average RMSD of less than 1.5 Å, with conformation 4 and conformation 3 occasionally slightly exceeding this value. In all cases RMSD remains below 2 Å. RMS fluctuations are similar for both parallel and anti-parallel simulations, and generally remain below a maximum of 2 Å. For the *h*DM2 protein in the arylamide complexes, there are regions of increased flexibility from residue 18–22, 44–47 and 69–77. The first two more flexible regions are common to all arylamide simulations and the p53 and small molecule simulations (Figure S6 in Text S1). These residues exist at the N-terminus of the high-affinity p53 peptide structure, with the glutamic acid in particular contacting the N-terminus of the helix. These residues also form a short loop between a pair of beta strands, which is likely to explain why the increased RMSF is observed in all structures from both the arylamide simulations and the initial MD simulations. The third flexible region in the arylamide system is between residues 69–77. Equivalent higher RMSF regions are not visible in Nutlin-2 and benzodiazepine simulations (Text S1, Figure S6c residues 79–87 or Figure S6d residues 69–77). The arylamide MD simulations exhibit RMSD less than 2.5 Å throughout the simulation, indicating that they are stable, and the RMSF, especially in the binding site, correlates well with the RMSF observed in the simulations of peptides and small-molecule binders (Figure S6 in Text S1). In summary, the similarly low RMSD from initial structures in the arylamide simulations as well as the mostly shared common areas of high and low fluctuation between simulations of the arylamides and structurally characterized binders suggest that the arylamides do indeed bind in the same pocket as the p53 peptide.

**Figure 5:**
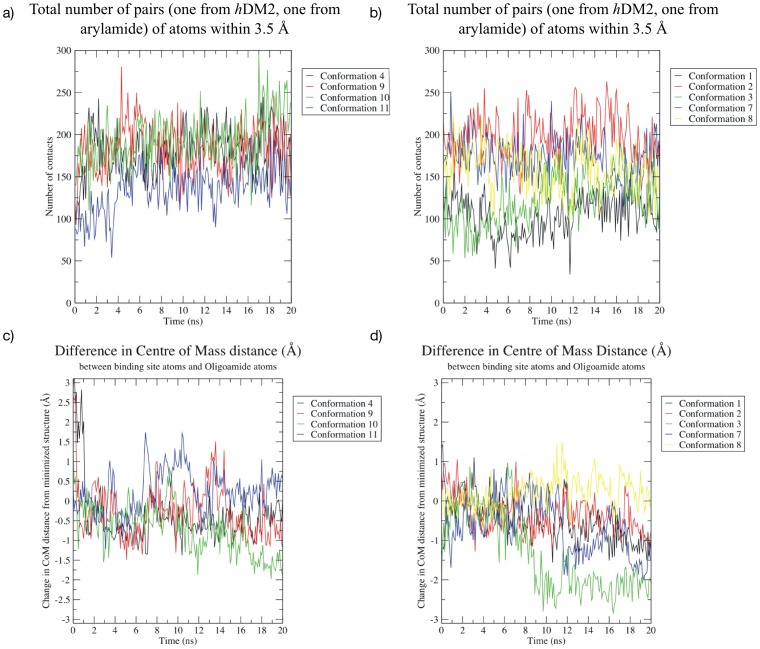
Time evolution of parallel (left) and anti-parallel (right) Phe-Nap-^i^Pr arylamide conformations. a) the number of intermolecular pairs of atoms between *h*DM2 and the ligand that are within 3.5 Å for parallel conformations; b) the number of intermolecular pairs of atoms between *h*DM2 and the ligand that are within 3.5 Å for anti-parallel conformations; c) difference in protein-arylamide center of mass distance (Å) from a minimized parallel starting conformation; and d) difference in protein-arylamide center of mass distance (Å) from a minimized anti-parallel starting conformation. The number of contacts tends to increase, indicating increasing stability of the bound arylamide, although there is wider variation in the number of contacts compared to the results presented in Figure S9 (Text S1). The hypothesis that an increasing number of contacts indicates increasing stability is supported by the decrease in the distance between the centers of mass of arylamide and *h*DM2 as the simulations progress, indicating a closer fit. It should be noted that decrease in the distance between centers of mass of ligand and protein is not always indicative of greater ligand burial therefore a system specific decision must be made before employing this analysis technique.

In the case of MD simulations of p53 peptides and known small-molecule inhibitors of *h*DM2, we observed that there was fluctuation but little deviation from the initial value of the center of mass distance between *h*DM2 and complexed ligand (Figure S8 in Text S1). This was likely due to the fact that the structures had already reached an equilibrium ensemble. Figure 5 depicts the number of contacts (defined as the number of intermolecular pairs of atoms between *h*DM2 and the ligand that are within 3.5 Å) and the differences in center of mass both of which provide a quantitative measure of stability for the arylamide compounds in the *h*DM2 binding site. Figure 5 shows results from parallel arylamide starting configurations on the left and anti-parallel arylamide starting configurations on the right. In the case of *h*DM2 bound to the Phe-Nap-^i^Pr arylamide, we know that not all docked compounds can be in stable equilibrium states since we have a variety of low energy docked complexes. Molecular dynamics simulations of *h*DM2-arylamide starting conformations identified by docking showed that the conformations tend to start with around 125 contacts and may take up to 5 ns to reach the equilibrium value of around 175 contacts (Figure 5). As the number of contacts increases, the center of mass distance decreases as the arylamide further explores tight fitting locations in the *h*DM2 binding site. Figure 5 does indeed show that in some simulations the average distance tends to decrease, a trend which is more pronounced in the anti-parallel simulations (Figure 5d). The decreased distance between centers of masses is particularly obvious in the case of conformation 3 and is mirrored by an increased number of contacts. Conformation 3 shows a decrease in center of mass distance of 2 Å (Figure 5d, green line) and additional increase in the number of contacting atoms (Figure 5b, green line), indicating that closer binding structures of the arylamide to the protein continue to be explored as the system equilibrates. The decrease in the center of mass is likely the result of free rotation of the ArCO bond for the arylamide N-terminal Phe group. Between 6 and 8 nanoseconds the Phe group rotated from a bound conformation in the pocket towards the solvent, between 8 and 9 nanoseconds the Phe group re-buried itself into the pocket more deeply.

Initial analysis of these MD simulations shows that the RMSD of each starting conformation relative to the docked structure is very similar for both parallel and anti-parallel simulations. Both parallel and anti-parallel conformations have similar deviation from the initial structure over time. The RMSD, RMSF, number of protein-ligand contacts and the protein-ligand center of mass distances from our MD simulations suggest that simulations of *h*DM2 with arylamides in the putative binding pocket behave similarly to those of *h*DM2 in complex with p53 peptides or small-molecule inhibitors. These dynamic structural similarities suggest that the simulations of arylamide compounds bound to *h*DM2 properly reflect the likely binding locations.

### Molecular Dynamics Simulations of Arylamide Compounds in Both Parallel and Anti-parallel Conformations are Consistent with Binding in the Putative Binding Site

When proceeding with molecular dynamics simulations of *h*DM2-arylamide conformations identified by docking, we choose to use one representative structure from each of a group of docked conformations. This structural diversity allows simulation of an ensemble which would not otherwise be obtained with a single starting configuration on the timescale of our simulations. For example, we did not see anti-parallel and parallel starting conformations interconvert on the timescale of our simulations. However, using five anti-parallel starting conformations and four parallel starting conformations, we were able to observe that some of these conformations begin to converge to a common group center for parallel and for anti-parallel simulations during the timescale of our simulations, resulting in partial convergence towards a consensus structure for parallel or anti-parallel conformations respectively. Not all arylamide compounds interconvert between clusters in our simulations, therefore we need to consider each of these conformations in any conclusions of equilibrium behavior. If we do not consider the individual starting conformations, molecular dynamics will not sample all states during the length of our simulations, which is of particular importance when considering thermodynamic properties of the *h*DM2-arylamide interaction. When arylamide conformations do not interconvert we can consider the individual simulations as sampling disjoint regions of phase space.

### Orientation and Positioning of Arylamide Compounds in the HDM2 Binding Pocket

**Figure 6:**
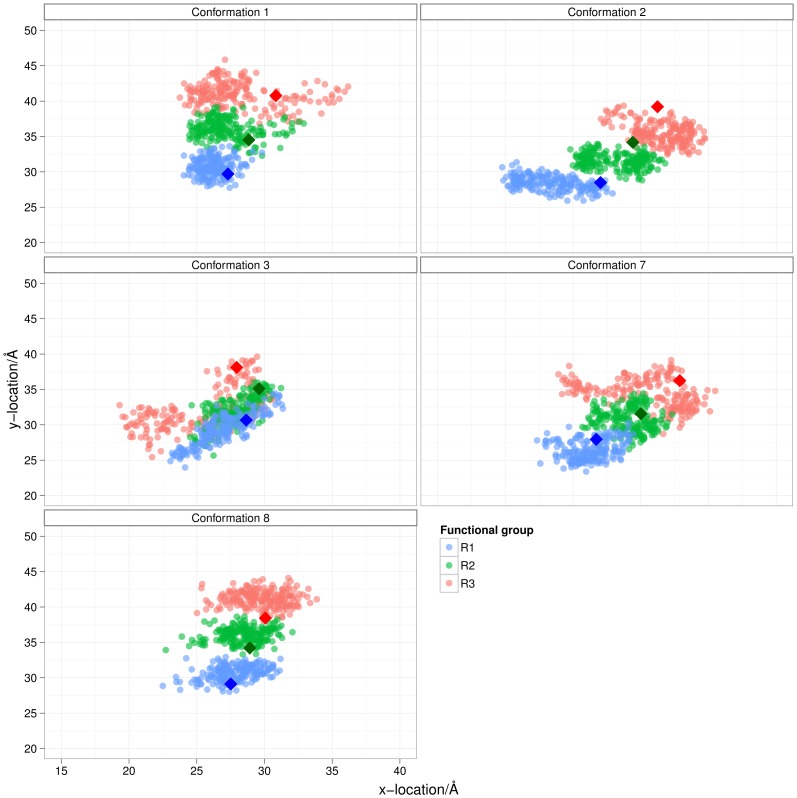
Orientation and positioning of arylamide compounds in the *h*DM2 binding pocket at 10 ps intervals. Four of five simulations sample in the same region of space, whereas simulation c does not. Ether oxygens from anti-parallel conformations of Phe-Nap-^i^Pr are projected onto a plane defined by Cα atoms from Tyrosine 56, Methione 62 and Valine 93. Data points are color-coded depending on which ether oxygen they belong to: R_1_ (Blue); R_2_ (Green); and R_3_ (Red). Data was plotted at 10 ps intervals starting after 4 ns of data collection. Values at t = 0 ps are plotted with diamonds. Graphs show images of starting conformation relative to the high affinity p53 helix and data from: a) conformation 1; b) conformation 2; c) conformation 3; d) conformation 7; and e) conformation 8.

**Figure 7:**
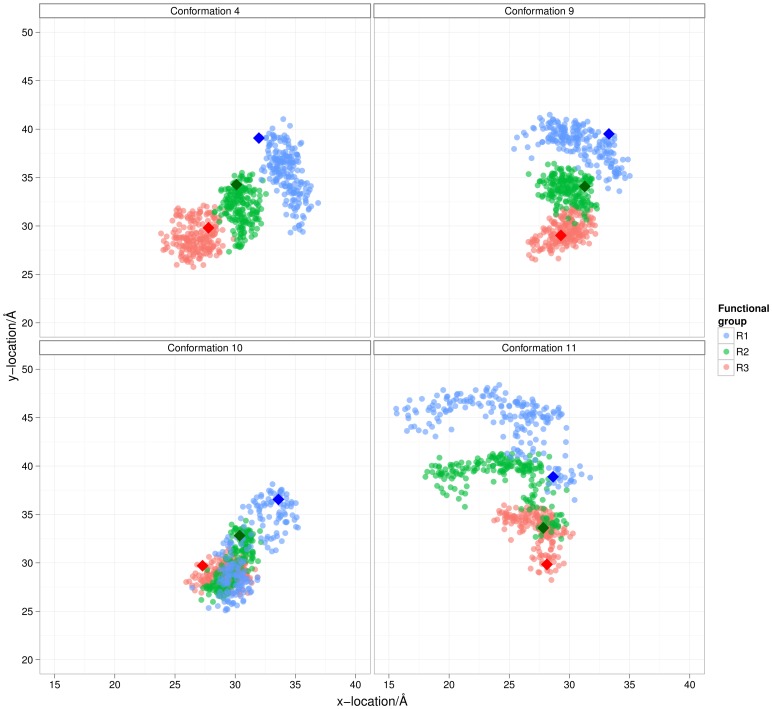
Ether oxygens from parallel conformations of Phe-Nap-Leu projected onto a plane defined by Cα atoms from Tyrosine 56, Methione 62 and Valine 93. Data points are color coded depending on which ether oxygen they belong to: R_1_ (Blue); R_2_ (Green); and R_3_ (Red). Data points were plotted at 10 ps intervals starting after 4 ns of data collection. Values at t = 0 ps are plotted with diamonds. Graphs show image of starting conformation and data from: a) conformation 4; b) conformation 9; c) conformation 10; and d) conformation 11.

We first investigate spatial sampling of the arylamides in the *h*DM2 binding pocket. The orientation and positioning of arylamide compounds in the *h*DM2 binding pockets is depicted in Figures 6 and 7. Here we show the projection of ether oxygen atoms from anti-parallel and parallel starting conformations onto a plane defined by three Cα atoms in the binding site (more detailed description of the projection is contained in the Methods section). The diamond points in the graph show the starting conformation. The N-terminal ether oxygen points are colored red, central ether oxygen points are colored black, and C-terminal ether oxygen points are colored violet.

The anti-parallel arylamide starting conformations sampled regions of the *h*DM2 binding pocket which overlap significantly with the region of space sampled by the R-groups of the high affinity p53 helix, as shown in Figure 6. In Figure 6b the arylamide is skewed such that the C-terminal ether oxygens are more positive in the x-direction and the N-terminal ether oxygens are more negative in the x-direction. Since the end-to-end distance of the arylamide compound stays approximately constant, the maximum y-distance explored is slightly less in the case of b with respect to a and e. Figure 6d shows similar behavior to what is observed in Figure 6b, although the ^i^Pr side-chain is significantly rotated out of the binding pocket at the start of the simulation due to a rotation about the ArCO bond of the central benzene ring. This is evident in the similar behavior of the R_1_ and R_2_ side-chains but the heavily skewed distribution of the leucine R_3_ side-chain. Conformation 3 in Figure 6c shows that the angle between the arylamide and the plane is much closer to 90° rather than a desired planar arrangement showing that the ligand is not bound in a similar manner to the previously discussed simulations. However, it does appear that this conformation samples the pocket quite differently to the other conformations and represents a completely different binding mode to that of the canonical helix form. Since we have no evidence to discount this conformation as a possibility we must explicitly consider this “perpendicular” configuration when proceeding further with simulations.

In contrast, parallel arylamide conformations do not converge structurally to a single ensemble. In the case of the parallel arylamide conformations in Figure 7, particularly in 7a and 7b, the C-terminus R_3_ of both simulations sample some of the same region of phase space. However, it appears that the N-terminus R_1_ of the two simulations explores a totally different region of space. This observation suggests that the docked conformation is actually a metastable state from which it decays into one of two or more stable states. The simulation depicted in Figure 7b shows the N-terminal phenylalanine remains in its rotated form (ArCO dihedral such that the R_1_ group is opposite the R_2_ and R_3_ groups) somewhat similar to the conformation in Figure 7d, while in Figure 7a, this dihedral relaxes such that R_1_, R_2_ and R_3_ exist on the same side.

### Most Side Chain Dihedrals are Well Sampled on the 20 ns Time Scale

Side chain dihedral angles can be some of the slowest observables to properly converge in molecular dynamics simulations, and observables such as binding free energies can significantly depend on dihedral conformations [49]. We therefore investigated the timescales of dihedral angle sampling in our simulations. We examined both dihedral angles from the *h*DM2 binding site side-chains and dihedrals present in the arylamide compound. We first examine which dihedral angles are likely to be well- or poorly-sampled on the timescales of our simulations. We then determine the relaxation time of the angle by fitting the autocorrelation function of a dihedral angle to a simple exponential model. The relaxation time corresponds to the average time the simulation would need to run for the dihedral angle to ‘forget’ information about its previous value. We can then use correlation time to guide whether we need to use several simulations with multiple starting configurations in the case of large relaxation times or whether a single starting conformation allows comprehensive sampling of the relevant regions of phase space.

**Figure 8:**
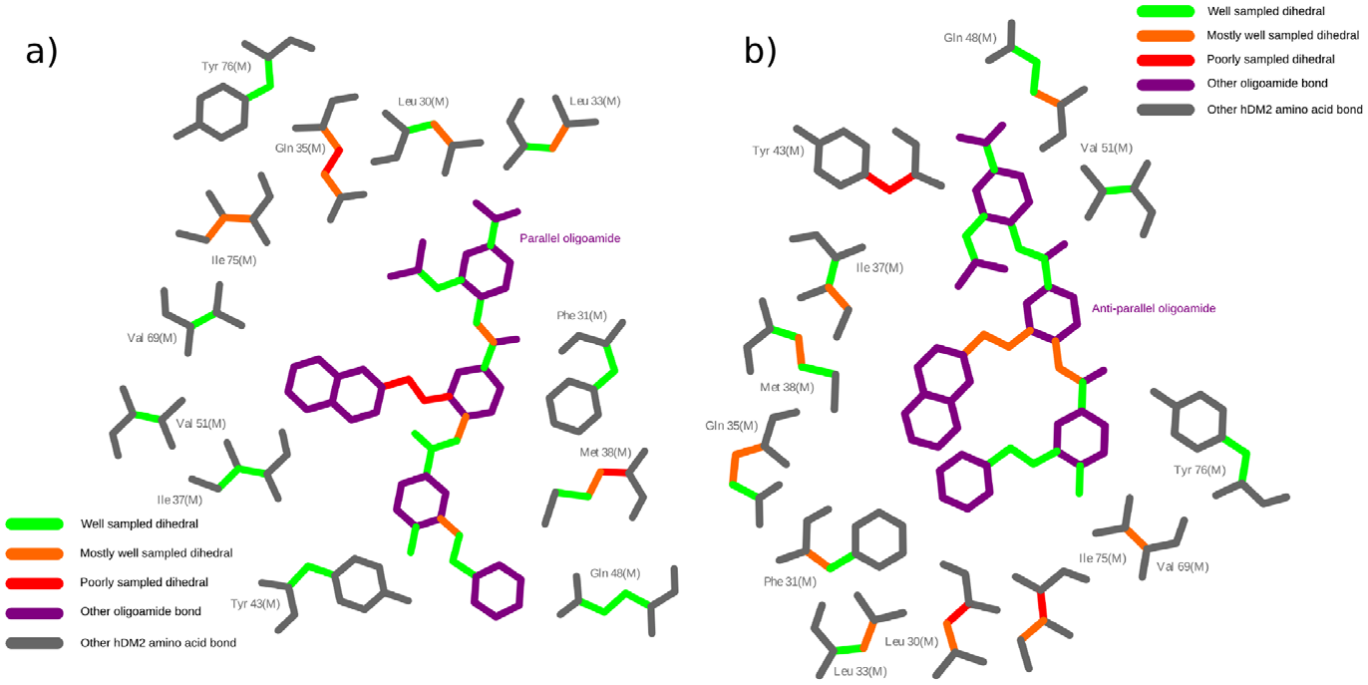
Dihedral sampling of binding site *h*DM2 residues and arylamide bonds. 2D representations of a) parallel and b) anti-parallel conformations of the Phe-Nap-^i^Pr arylamide in the *h*DM2 binding site are produced using Ligplot [51]. Amino acid backbone residues and bonds with no dihedrals are shown in grey, while arylamide torsions without flexible dihedrals are shown in purple. Rotatable bonds are colored according to our sampling criteria with: green (well sampled); orange (well sampled in all but one simulation); red (poorly sampled across simulations). It is clear that many dihedral angles are well sampled, but some are not, indicating that multiple starting configurations must be used for calculating thermodynamic properties of the system.

The dihedral distribution is plotted in Figure 8 for each of the simulations and compared to the other distributions. The dihedral is classified as ‘well sampled’ if all the distributions are in agreement. The dihedral is labeled as ‘mostly well sampled’ if only one of the distributions differs significantly from the others. The dihedral is labeled as ‘poorly sampled’ if more than one distribution contains a region that differs significantly between simulations, which implies that more than one simulation does not sample a possibly important region of dihedral space. In such cases, it will significantly help to use multiple starting conformations to estimate thermodynamic properties as rotameric states that are not sampled in a single simulation may contribute significantly to the free energy of interaction [45].

The majority of dihedral angles within the arylamide compounds in both parallel and anti-parallel starting conformations are well sampled. Figure 8 shows a representation of the parallel and anti-parallel Phe-Nap-^i^Pr arylamide compounds. Bonds with dihedral angles are shown in bold, with the quality of sampling of the angle denoted by color. From a total of 16 dihedral angles investigated, well-sampled dihedral angles (green) are observed in 10 parallel and 11 anti-parallel dihedral angles. There are three mostly well-sampled dihedral angles (orange) from parallel conformations and five mostly well-sampled dihedral angles in the anti-parallel simulations. There are no poorly sampled dihedral angles (red) in the anti-parallel conformations, while the parallel conformations have poorly sampled dihedral angles for the three χ angles for the bonds attaching the 2-napthalene group. In both cases, there are no aromatic residues that might restrict dihedral sampling due to intermolecular π–π stacking effects, assuming that the arylamide binding mode somewhat mimics that of the p53 peptide (Figure S10 in Text S1).

Using the same three category classification scheme as used to classify amino acid side chain dihedral angles for the arylamide compound dihedral angles, we see in Figure 8 that dihedral angles are often well sampled or mostly well sampled in both parallel and anti-parallel simulations. In fact, from a total of 25 dihedral angles we see only two poorly sampled dihedrals in the case of simulations of the parallel binding configurations and only four poorly sampled dihedrals in the case of simulations of the anti-parallel binding configurations.

The relaxation times for *h*DM2 binding site residue dihedrals of both the parallel and anti-parallel binding configurations are often longer than the total length of the simulation (Text S1, Figure S11 and Figure S12). The long relaxation times observed for some protein side chain dihedral angles means that some side chain conformations are trapped, requiring that we consider multiple simulation starting points when simulating these structures.

**Figure 9:**
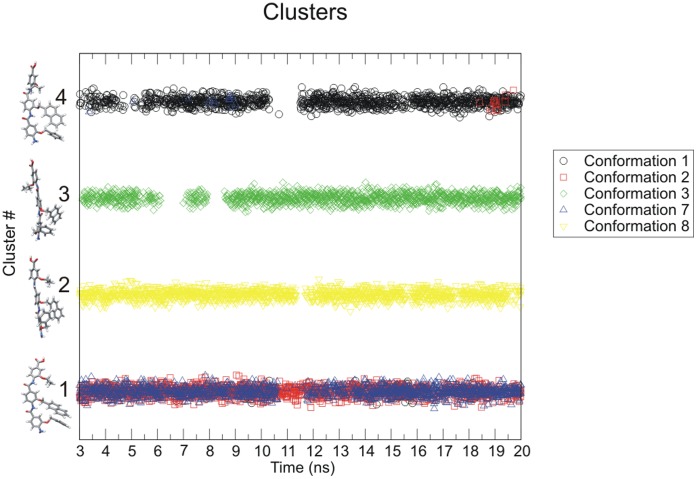
Occupancy of the top four anti-parallel clusters. Color coded by starting conformation during the final 17 ns of the simulation. Initial conformations 1, 2, and 7 interconvert between clusters #1 and #4.

We use cluster analysis to track those conformations of the arylamide compound occurring during our simulations and observe which structural clusters interconvert on the timescale of the simulations. Snapshots were taken every 10 ps for the final 17 ns of simulation, resulting in a total of 8,500 anti-parallel conformations of arylamides. When clustering anti-parallel arylamide conformations we observe nearly 3000 members of the most populated cluster, 1500 for the second most populated, approximately 1400 for the third most populated and just short of 1250 for the fourth most populated. The fifth most populated cluster has fewer than 500 members (Figure S13 in Text S1). Although there are 33 clusters of anti-parallel conformations in total, 84% of conformations are contained in the four top ranked clusters. Figure 9 shows which of the top four clusters (representative cluster members shown in stick representation) are populated by arylamide conformations from each of the five simulations as a function of time. We see that cluster four is visited predominantly by the simulation with starting conformation one, however, it is also visited by simulation 2 and 7. Thus the three starting conformations 1, 2 and 7 can likely be treated with a single simulation, since each of these states is likely to be visited on the 20 ns time scale. For simulations that are not converged one may expect different average values when calculating using conformation 1, 2 or 7 as a starting point, however, for a simulation that approaches thermodynamic convergence one would expect average values to be comparable as the states can interchange on these simulation timescales. In the case of cluster 2 and cluster 3, we do not see any inter-conversion with other highly populated clusters, thus 20 ns simulations must consider using conformation 3 and 8 as starting points for simulation in addition.

**Figure 10:**
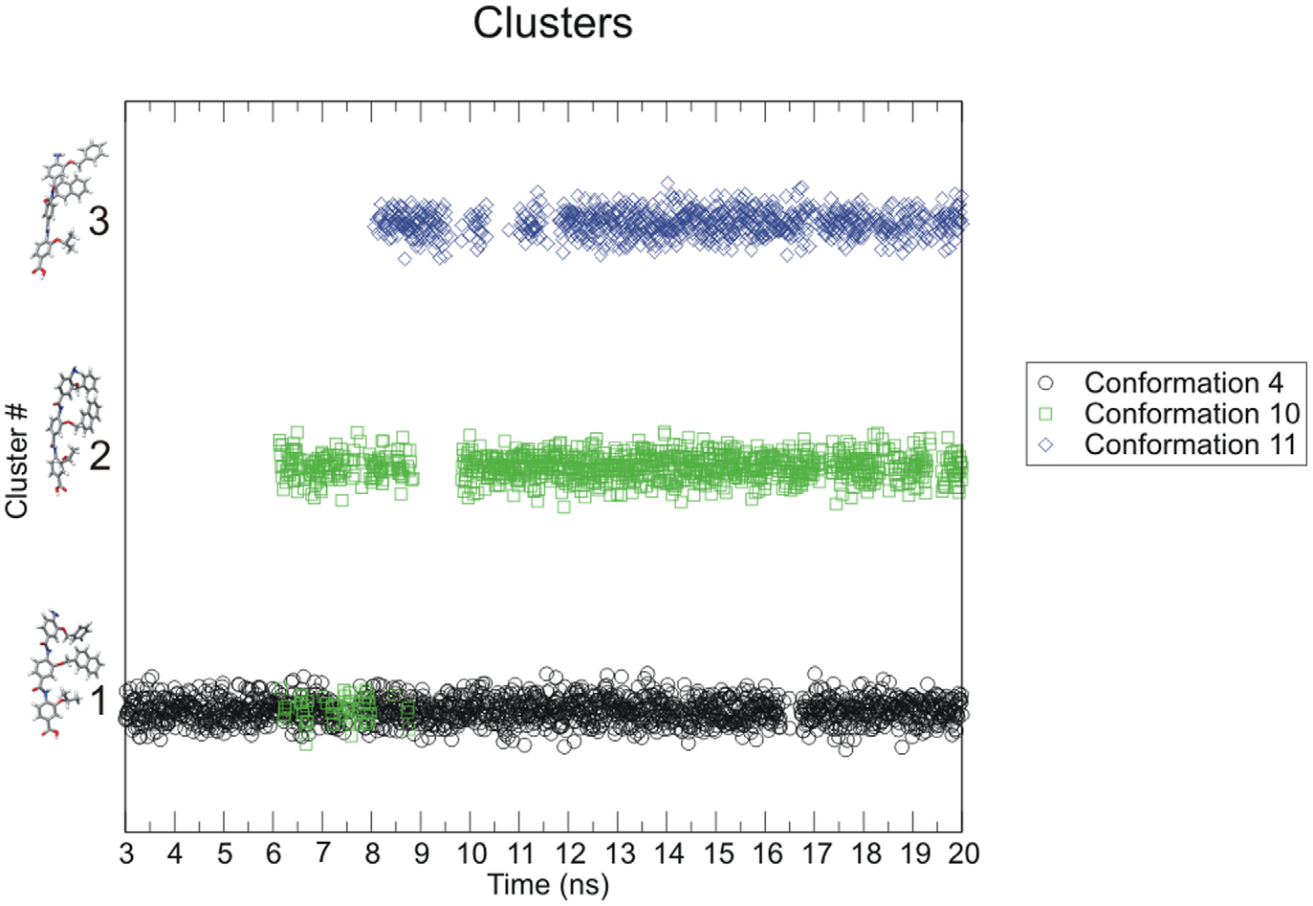
Occupancy of the top 3 parallel clusters. Color coded by starting conformation during the final 17 ns of the simulation. Inter-conversion between clusters #1 and #2 is observed by conformation 4 and 10.

When clustering parallel simulations we observe approximately 1700 members in the most populated cluster, slightly more than 1000 in the second most populated and just short of 900 in the third most populated cluster. There are 27 clusters in total with more than 50% of conformers contained in the top 3 clusters. Parallel arylamide conformations show some inter-conversion between low population clusters, but not significant inter-conversion between the most occupied clusters. In Figure 10 we show the representative member of each of the top 3 clusters from parallel starting conformations. In this case, it is immediately obvious that both clusters #2 and #3 are not populated during the first 6 ns and 8 ns of the simulations. However, after this time they begin to be occupied far more often, implying that the ligand conformation converges towards multiple structural clusters during the simulation. For simulations of anti-parallel conformations we can conclude that there is a reasonable amount of inter-conversion between clusters #1 and #4, hence it may be acceptable to choose only a single representative to sample these states sufficiently. However, for simulations of parallel conformations we conclude that several favored conformations are identified (Figure 10), although inter-conversion between these clusters does not occur on the 20 ns time scale, suggesting that multiple starting conformations must still be considered for further study.

## Conclusions

The main aim of these experiments was to test the hypothesis that arylamide alpha helical mimetics bind to the putative binding site of *h*DM2 using docking and molecular dynamics simulations. This detailed structural analysis of the binding site also helps establish the structural properties of *h*DM2-arylamide binding for use in further studies such as binding free energy calculations [50]. These results are especially important for synthetic chemists who want to design new side chains for the arylamide compounds in order to improve selectivity and affinity for the *h*DM2-p53 interaction and also for any further studies of the *h*DM2-p53 interaction using computational docking or molecular dynamics techniques.

In this study, we have used a variety of techniques including docking and molecular dynamics simulations in order to investigate helix mimetic arylamide binding to *h*DM2. We showed that the conformer generation program OMEGA is able to predict the arylamides’ conformations. The widely used force fields of GAFF and Autodock do not accurately predict the conformations of the arylamides, while MMFF94 (used in Omega) could produce plausible low energy arylamide conformers. We also showed that the GAFF and Autodock force fields can be modified to improve their performance in order to accurately reproduce the behavior of arylamide backbones.

Simulations of *h*DM2 bound to arylamides are comparably stable to those of *h*DM2 in complex with p53 peptides or previously identified small-molecule inhibitors when using measures such as RMSD, RMSF, number of atomic contacts or protein-ligand center of mass distance. This behavior validates our choice of a force field designed to both simulate the properties of the protein well and be compatible with the GAFF force field, allowing the simulation of a large number of possible arylamide side-chains. Alternative force fields, not tested in our study, may be suitable for the study of arylamides in combination with proteins. However, this experience suggests similar calculations to determine whether an alternative force field sufficiently reproduces known experimental observations should be performed. This finding also highlights the need to be cautious when applying such ready-made force fields to atypical ligand chemistries. Researchers should be prepared to perform QM in many cases. The ability to compare with structural information on the ligand in solution may also be necessary in the absence of structural information about the bound complex.

We discovered that there are two classes of arylamide conformation identified by docking which are stable in molecular dynamics simulation: parallel and anti-parallel. Previous docking studies only identified the parallel conformation [29]. However, docking cannot distinguish which of these classes might be more stable. It is likely, of course, that both are stable, and the closeness in the stability suggests that it might be possible to bias one conformation over another by altering the chemical groups present on the arylamide side-chains. Such antiparallel configurations must therefore be considered in future arylamide inhibitor design. The possibility of steric clashes between parallel configurations and the *h*DM2 protein suggests that it may be possible to design inhibitors to adopt only parallel or anti-parallel configurations by either promoting or reducing this clash as desired by modification of the arylamide N-terminus. The compound studied in this work, and related compounds studied in work by Plante *et al.* are unlikely to have constituent side chains that are sufficient to bias the orientation to consistently adopt only parallel or anti-parallel conformations. Future design of arylamide compounds that target charged residues at either end of the helix binding cleft might be able to exploit charge complementary to bias towards either parallel or anti-parallel binding.

Our procedure of extensive docking, combined with several shorter molecular dynamics simulations identified several putative metastable conformations, showing that single simulations of 20 ns are not necessarily sufficient to capture the full functional flexibility of *h*DM2-arylamide binding. In the section *‘Orientation and positioning of arylamide compounds in the hDM2 binding pocket’*, and using cluster analysis, we identified two putative metastable conformational states, based on the fact that of nine starting conformations, five converged to sample the same two distinct conformational ensembles. Inter-conversion between states, identified using cluster analysis, during the 20 ns simulations indicates that a single starting conformation cannot be used to simulate the equilibrium properties of the system. Because arylamide clusters do not always inter-convert on the timescale of our simulations, multiple starting conformations must be considered when investigating equilibrium properties of the system.

We have also presented a novel projection method to characterize the spatial sampling in the *h*DM2 binding site, which could be used in other protein binding studies. This projection technique allows us to show how the arylamide compounds sample the *h*DM2 binding site in space and time. In combination with cluster analysis of arylamide conformations, this procedure can characterize the convergence of arylamide conformational ensembles. Such quantitative structural equilibration analysis techniques are currently rarely performed for ligand binding. These techniques could easily be applied as a straightforward method to determine structural sampling in other protein-ligand systems, allowing researchers to visualize the region of the binding pocket that is sampled by different functional groups of a ligand.

When investigating the specific arylamide in our study we showed the necessity of several techniques for improved characterization of binding sites, such as the need to validate sampling parameters used in docking, the use of clustering to identify metastable states, and the need to characterize the spatial sampling of the binding site. These techniques are likely necessary for simulating a diverse range of arylamide and other nonstandard chemical compounds, and more generally for simulating binding pockets and designing drugs. The care required to perform these sorts of analysis to truly identify physically reasonable binding sites present a cautionary tale for the use of molecular simulations to identify bound complexes of novel classes of compounds.

## Supporting Information

Text S1 **Supporting Information. **A Supporting Information document is available in PDF format. It contains Supporting Figures (Figures S1-S19), Methods and Tables from *h*DM2 binding site analysis, docking calculations, arylamide charge derivation (see Figure S14, Figure S15, Figure S16, Figure S17, Figure S18, Figure S19) and *h*DM2/ arylamide conformational analysis.
(PDF)

File S1 **Arylamide Conformations Zip.** A zip file containing PDB coordinates of the hDM2 structure 1T4F used for docking and simulation, the docked arylamide conformations, and Gromacs .itp files containing the arylamide bonded and non-bonded parameters used for molecular dynamics simulations is available.
(ZIP)
