# Supplementary material for: Modeling of Arylamide Helix Mimetics in the p53 Peptide Binding Site of hDM2 Suggests Parallel and Anti-Parallel Conformations Are Both Stable
Source: PLoS One. 2012 Aug 20;7(8):e43253. doi: 10.1371/journal.pone.0043253 (PMC3423354; doi:10.1371/journal.pone.0043253)
Supplement: Text S1 — Supporting Information. A Supporting Information document is available in PDF format. It contains Supporting Figures (Figures S1-S19), Methods and Tables from hDM2 binding site analysis, docking calculations, arylamide charge derivation (see Figure S14, Figure S15, Figure S16, Figure S17, Figure S18, Figure S19) and hDM2/ arylamide conformational analysis. (PDF) [file pone.0043253.s001.pdf]

Supporting Information

Modeling of Arylamide Helix Mimetics in the p53 Peptide Binding Site of *h*DM2 Suggests Parallel and Anti-parallel Conformations are Both Stable

Jonathan C. Fuller, Richard M. Jackson, Thomas A. Edwards, Andrew J. Wilson and Michael R. Shirts

Table of Contents

Docking Images (Autodock): Pages 2-3

Geometric matching: Page 4

Superposition method: Pages 5-6

Binding Site Analysis: Pages 7-8

Docking Image (FRED): Page 9

MD simulations of *h*DM2 bound to: p53 peptides, Nutlin-2 and benzodiazepinedione: Pages 10-17

Binding site residues figure: Page 18

Autocorrelation bar-charts: Pages 19-20

Cluster bar-charts: Page 21

Charge Calculation Method: Pages 22-35

References: Page 36

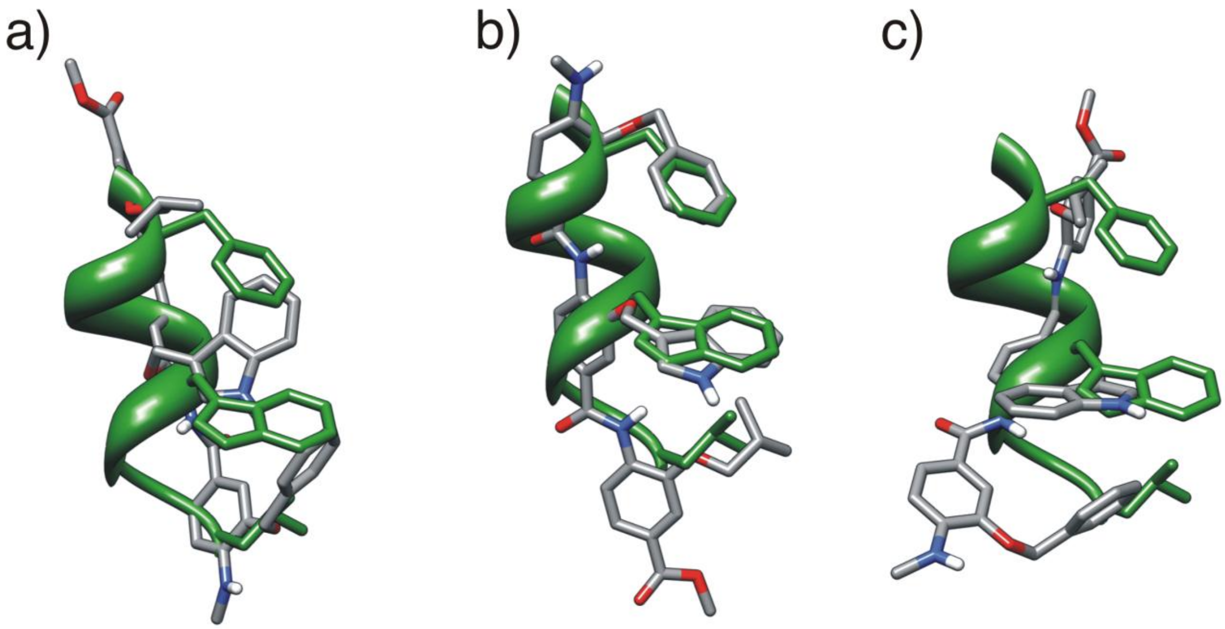

Figure S1: Representative structures from a preliminary docking screen that were used for initial MD studies (atom colored), shown relative to high affinity helix from 1T4F. Representatives from: a) Cluster 1, representative 6; b) Cluster 2, representative 1; and c) Cluster 3, representative 6. Figures were generated using the matchmaker function from Chimera to superpose hDM2 from docked conformations to hDM2 from 1T4F.[3]

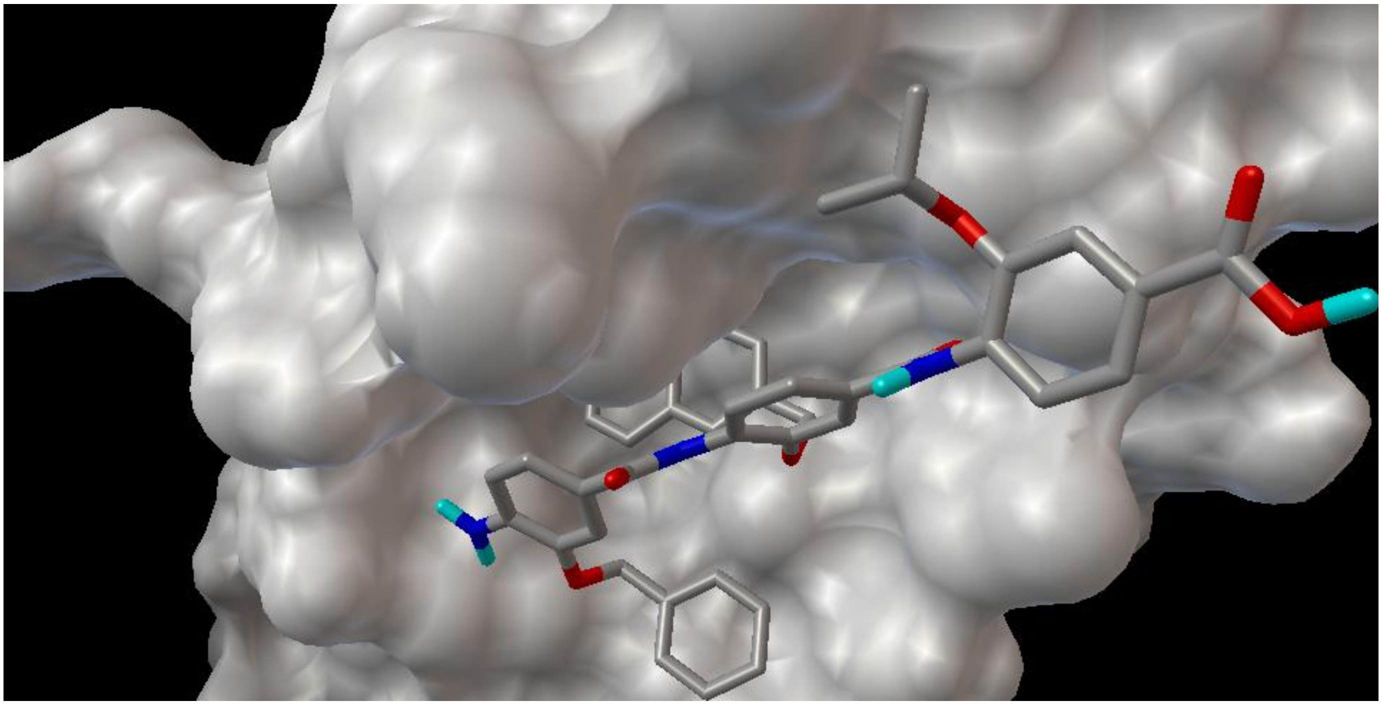

Figure S2: A representative of the second-most highly populated cluster for an Autodock experiment where the ArNH torsion was not restricted. Image shows the non-planar arrangement of the amide NH group and the ether oxygen.

Geometric matching

A geometric hashing algorithm was used to superpose atoms from arylamide compounds that had been generated by OMEGA. The method has been described previously,[1] but is described here briefly for completeness. Triplets of atoms that by definition form a triangle are generated for the database molecule (p53 peptide) and query molecule (arylamide compound). All possible pairs of triplets, where each pair consists of a triplet from both query and database molecules, are compared. All triplets with the same atom at each triangle vertex and similar distances between vertices are treated as a match. The resulting triplets then define a rotation and translation matrix which will map the query molecule onto the database molecule. At this point the number of coincident atoms can be determined, and the transformation which provides the largest number of coincident atoms is treated as the best match.

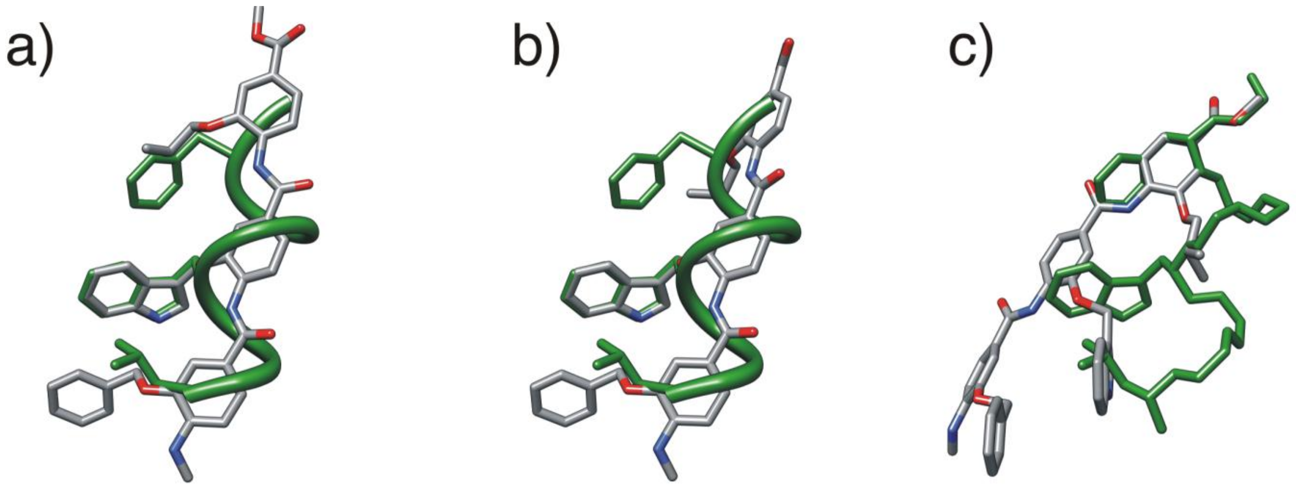

Figure S3: Arylamide compounds shown colored according to atom type superposed onto the binding Phe-Trp-Leu residues, and backbone atoms from the high-affinity p53 helix shown in dark green. All compounds shown are oriented in the anti-parallel conformation with: a) showing a good match; b) a reasonable match; and c) a poor match which would sterically clash with the hDM2

Superposition Method

Since we are examining the hypothesis that side-chains from synthesised oligamide compounds directly mimic the side-chains from the p53 helix that are known hotspot residues in the *h*DM2 interaction, it is reasonable to assume that a simple method for generating starting conformations for free energy calculations is to simply overlay the arylamide onto the p53 helix such that the side-chains mimic the *h*DM2-p53 interaction as closely as possible. We used the GH8 program to superpose arylamide conformers generated using the OMEGA program onto the high-affinity p53 helix. We observed that as this was carried out there was a heavy bias to matching arylamide atoms to the helix side-chain atoms that are known to be less energetically important for the interaction. As a result we ran the superpositions again using only the side-chain atoms from the Phe-Trp-Leu residues. We now observed that whilst we no longer had the problem of matching side-chains to the wrong region, we lost information from the peptide about the preferred orientation of the side-chains with respect to the protein. That is, while we might match atoms from the arylamide side-chains to those of the peptide well, we might then arrange the arylamide backbone where the *h*DM2 protein would normally exist. We struck a balance by using only the peptide backbone atoms and all of the atoms from the Phe-Trp-Leu residues.

Representative structures from the method are shown in Figure S3, with Figure S3a showing a fairly successful match. In this case, the tryptophan rings are matched extremely well, but the method only identified anti-parallel conformations. Figure S3b shows a similarly successful match, which is slightly worse due to the fact that the Leu residue is twisted out of alignment from the Phe residue of the peptide. Figure S3c shows a bad match which would result in a steric clash with the *h*DM2 protein. This is similar to many of the matches that were observed in the previously described superposition of arylamide compounds onto only Phe-Trp-Leu residue side-chain atoms.

We observed several issues with using superposition methods in the context of this system. The first is that it is difficult to score the results in such a way that it would be possible to prioritise those that are

more likely to be observed in reality. For example, 15 atoms are matched in both figures S3a and S3b, but when looking at the results a) is clearly a better match. Additionally Figure S3c matches 14 atoms, only one fewer than S3a and S3b, but does not match the side-chain atoms very well and would have problems with steric clashes with the *h*DM2 protein atoms. While this would be acceptable for setting up a single system, this method would not be appropriate for a larger scale system where several different arylamide compounds are investigated. Additionally it is not clear how well this method would fare when applied to arylamide compounds that have side-chains designed to bind *h*DM2 with higher affinity, whilst perhaps having different molecular shapes. For example, a napthyl ring instead of a tryptophan ring is likely to score less well even if all of the non-ring atoms are located in exactly the same place. The final consideration is that the method is very reliant on the quality of the conformations used in the method, and while it appears that OMEGA is successful in generating good conformers, in the absence of a sensible scoring scheme a large amount of manual inspection is required that is unfeasible for high throughput applications.

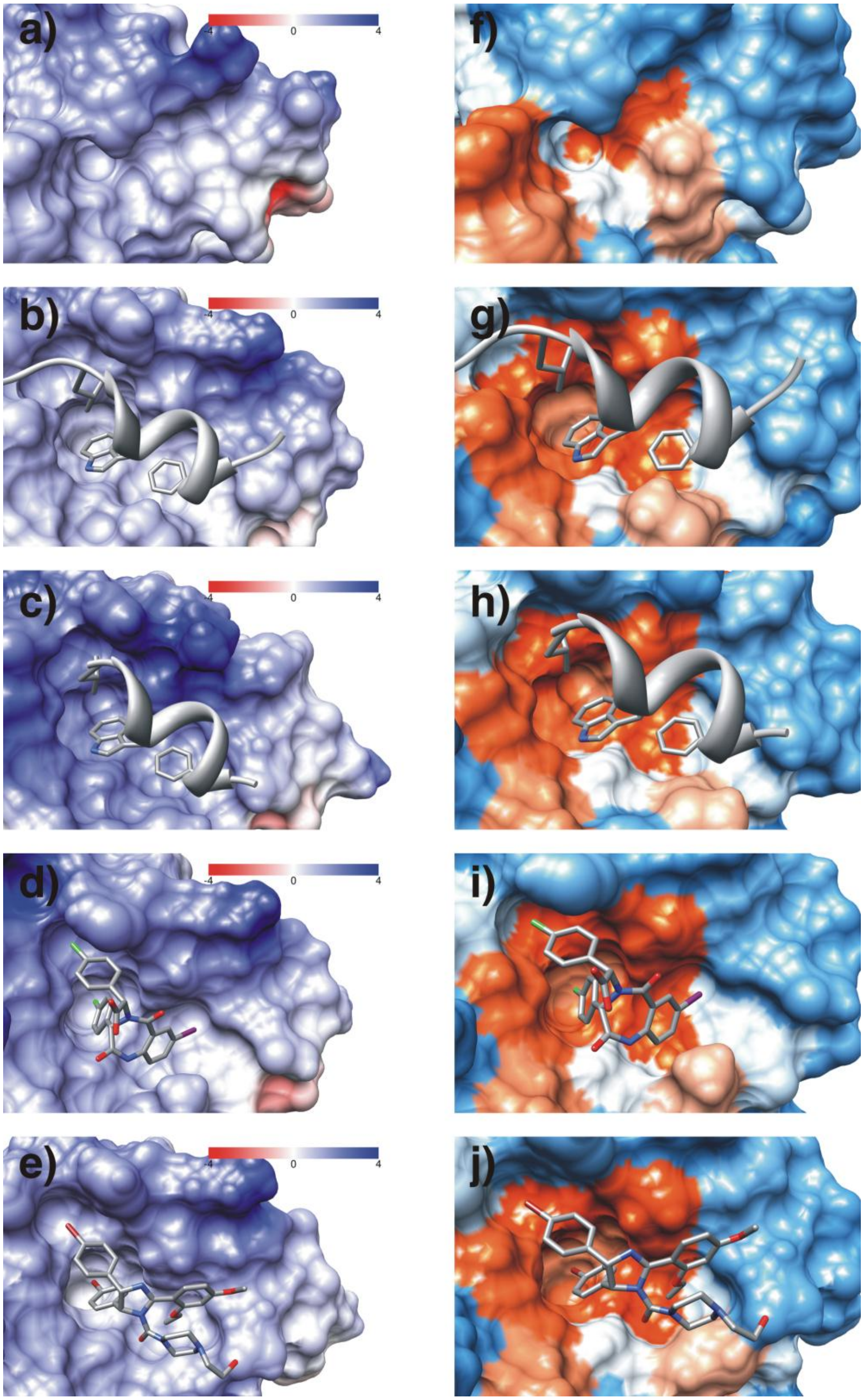

Figure S4: The hDM2 binding pocket shown with electrostatic surfaces (red - negative charge, blue - positive charge) a)-e) and hydrophobic surfaces (blue - hydrophillic, white - no preference, orange - hydrophobic) f)-j). a/f) hDM2 apo (1Z1M); b/g) hDM2 wild type p53 (1YCR); c/h) hDM2 high affinity p53 (1T4F); d/i) hDM2 Benzodiazepinedione (1T4E); e/j) hDM2 Nutin-2 (1RV1). Images produced using Chimera, electrostatic surfaces calculated using Delphi.

Binding Site Analysis

Electrostatic surfaces

Electrostatic surfaces were calculated using DelPhi V. 4 Release 1.1,[2] with computations carried out through the DelPhi controller module of UCSF Chimera.[3] An interior dielectric of 2.0, an exterior dielectric of 80.0 and Debye-Huckel boundary conditions were used in the calculation. Results were visualised using UCSF Chimera version 1.4 on the Mac OS X operating system.

Hydrophobic surfaces

Hydrophobic surfaces were generated using the hydrophobic surface preset from UCSF Chimera version 1.4 on the Mac OS X operating system.[3] Residues are coloured according to the Kyte-Doolittle scale, with blue showing the most hydrophillic residues, white showing a value of 0.0 and orange showing the most hydrophobic residues.[4]

hDM2 Binding Pocket Hydrophobicity

We can see in Figure S4 that the pocket is particularly hydrophobic, as is mentioned by Grasberger et al.[5] Additionally, we can see from Figure S4 that the pocket doesn't carry a strong electrostatic charge. Hydrophobic pockets are often difficult to develop compounds that bind with a high degree of specificity. Additionally binding affinity can sometimes be gained by the use of halogenated functional groups, such as the chlorophenyl rings seen in Benzodiazepine compounds.[5] The main issue with these elements is the decrease in solubility that is observed both experimentally and through QM/MM studies.[7] Additionally, halogenated drug compounds often show undesirable ADMET properties such as accumulation in fat tissue.

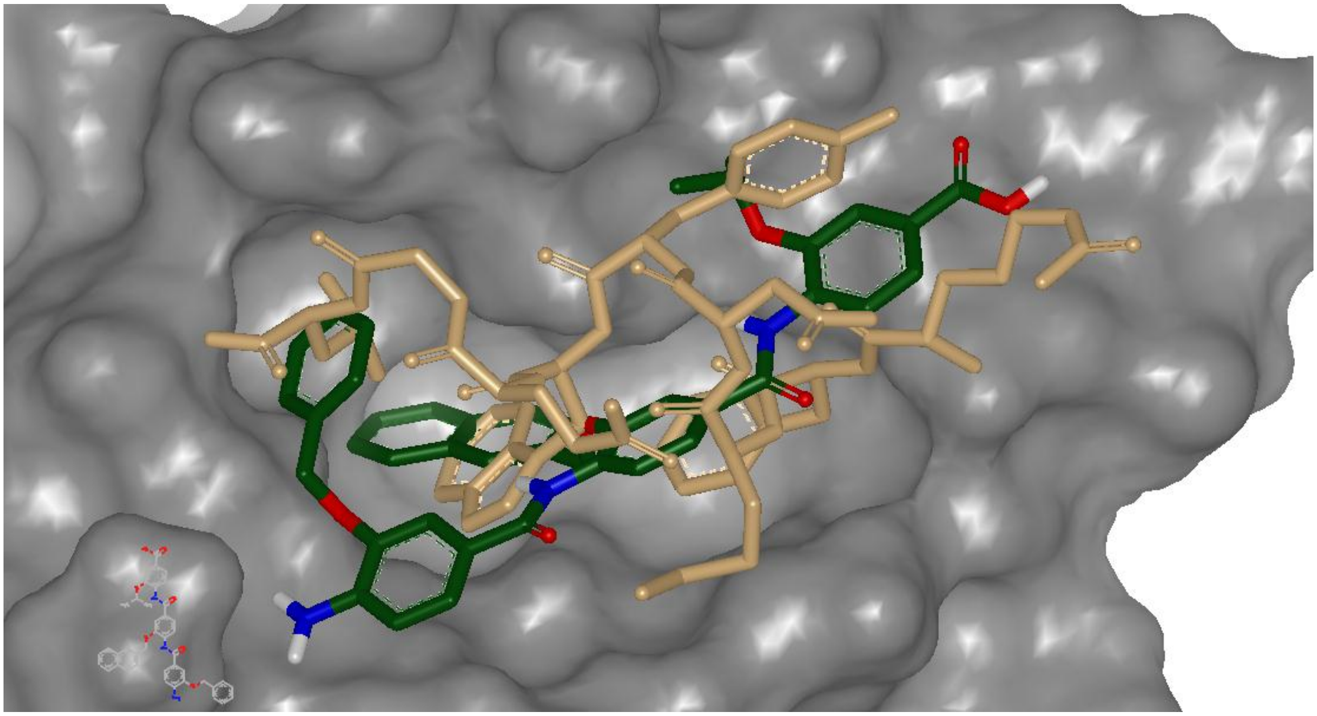

Figure S5: Best pose from FRED using the Chemgauss 3 scoring function (green, red, blue and white coloured atoms) compared to the Phe-Trp-Leu high-affinity p53 helix. hDM2 molecular surface shown in grey. Note the tyrosine ring from the p53 helix (beige) towards the top right hand corner of the figure.

Molecular dynamics simulation of p53 peptides and small-molecule inhibitors of hDM2

We used the crystal structures of *h*DM2 bound to p53 peptides and small-molecule inhibitors as a starting point for molecular dynamics simulations.

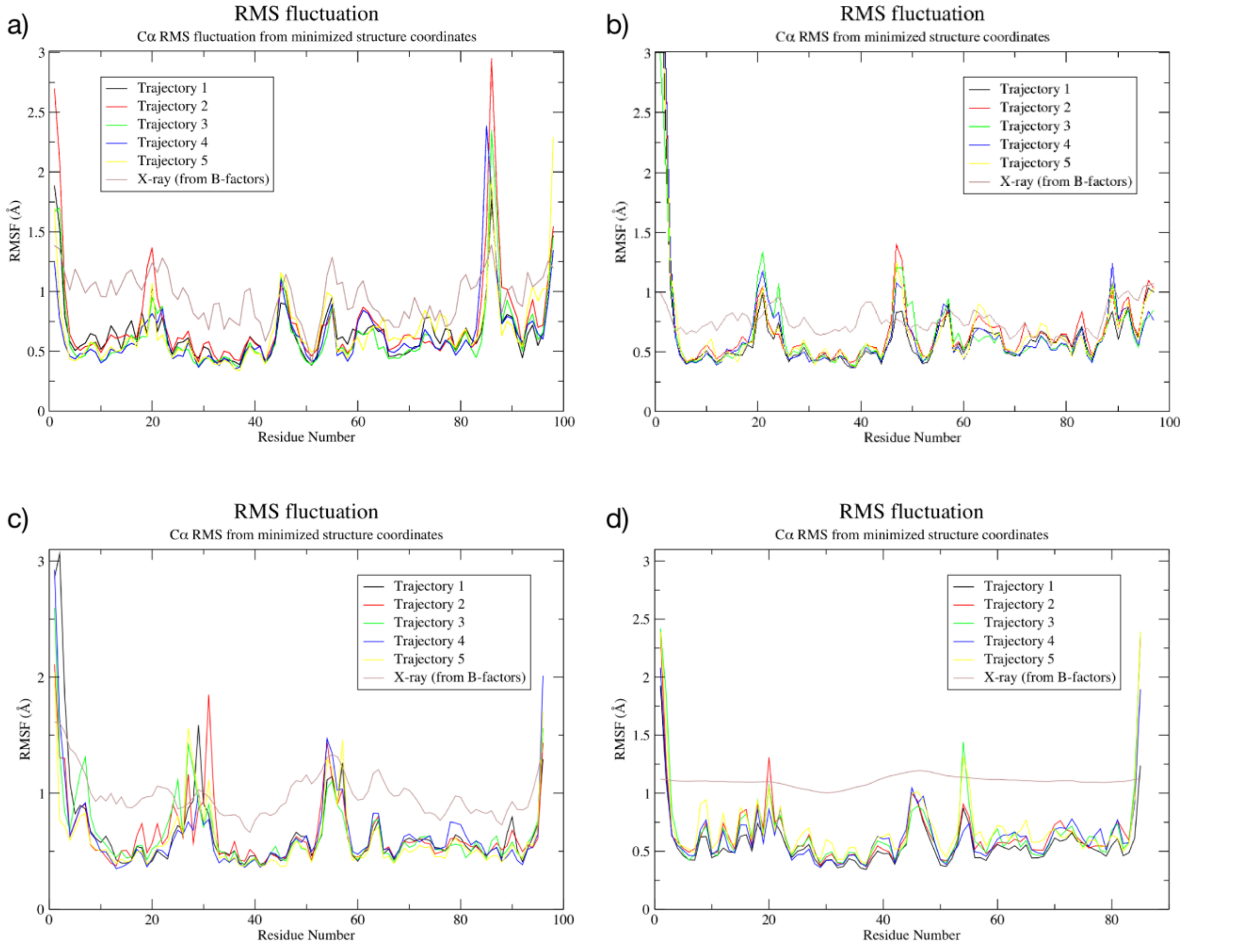

Figure S6: RMS fluctuation compared to experimental B-factor (as specified in the corresponding PDB file), using the relationship $RMSF = \sqrt{3B/8\pi^2}$ for 5 replicates of hDM2 simulated for 10 ns in complex with[6]: a) wild-type helix (1YCR); b) high-affinity helix (1T4F); c) benzodiazepinedione (1T4E); and d) Nutlin-2 (1RV1). B-factors are not inconsistent with calculated RMSFs, indicating stable simulations that have similar dynamics to the crystal structures. However, it should be noted that the crystal structure resolutions are only moderate (2.6, 1.9, 2.6 and 2.3 Å respectively), and the B-factors may therefore contain contributions from static disorder.

RMS fluctuation of protein: We observe the RMSF of the residues is comparable between simulations and correlates with experimental B-factors. When discussing the RMSF of residues we omit the first and last residues, since they are not restrained by the motion of surrounding residues and are therefore much freer to move than one would expect to observe in most crystal structures. Figure S6a is the RMSF plot for hDM2 bound to wild-type p53. We see excellent agreement in the RMSF between simulations, and furthermore the simulations correlate with the RMSF calculated from the experimentally determined B-factor. The maximum values from the RMSF are between 2.5 Å to 3 Å, which is approximately double the RMSF calculated from the experimental B-factor (using the formula $RMSF = \sqrt{3B/8\pi^2}$).[6] We observe similar results in Figure S6b in the case of *h*DM2 bound to high-affinity p53. Each of the five replicates has consistent values of the RMSF during the simulation, showing that our simulations are reproducible. Figure S6c shows the RMSF for hDM2 bound to benzodiazepinedione, which shows a distribution of RMSF values that closely follows the RMSF calculated from the experimental B-factor. Figure S6d shows that the RMSF for *h*DM2 bound to Nutlin-2 calculated from the experimental B-factor is nearly flat with a value of approximately 1.2 Å. This perhaps indicates that the experimental B-factors from this structure might be less reliable than for the other three structures or that the B-factor only corresponds to the crystal environment, since our simulations show reproducible flexibility in certain regions of the chain that one would expect to be reflected in the experimental B-factor. The simulations of Nutlin-2 have RMSF remaining below this 1.2 Å value except for slight deviations at two residues.

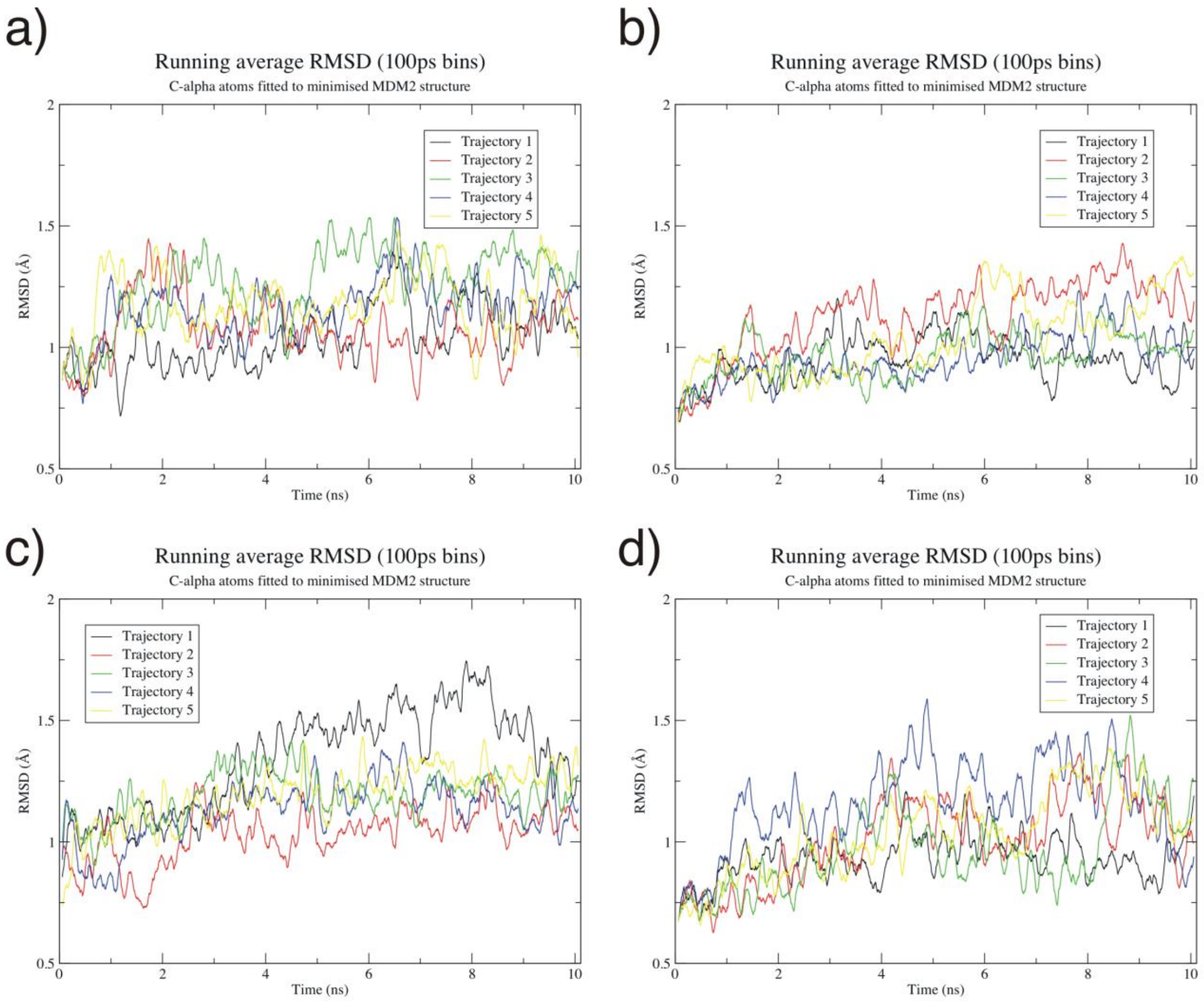

Figure S7: Running averages (100 ps window) of the RMSD (Å) for 5 replicates of hDM2 simulated for 10 ns in complex with: a) wild-type helix; b) high-affinity helix; c) benzodiazepinedione; and d) Nutlin-2. RMSD of hDM2 bound to p53 peptides or small-molecule inhibitors does not increase significantly over time after the first 4 ns.

RMSD from initial structure: The RMSD of simulations of p53 peptides and small-molecule inhibitors of hDM2 in complex appear to equilibrate to constant values within a few nanoseconds. The RMSD between these structures and their minimized structures is shown in Figure S7, with *h*DM2 in complex with: a) wild-type p53 helix; b) high-affinity p53 helix; c) benzodiazepinedione; and d) Nutlin-2. In all

but two cases the RMSD never exceeds 1.5 Å. We consider our simulations to be stable if RMSD < 2.5 Å. In the two cases where RMSD does exceed 1.5 Å it remains well below the 2.5 Å cutoff and returns to less that 1.5 Å. These two cases are trajectory 1 in the benzodiazepinedione simulation (Figure S7) and trajectory 4 in the Nutlin-2 simulation (Figure S7). The stability and reproducibility of the simulations is evident in Figure 7, as shown by the low RMSD from the starting structure and the small variation in RMSD between simulations.

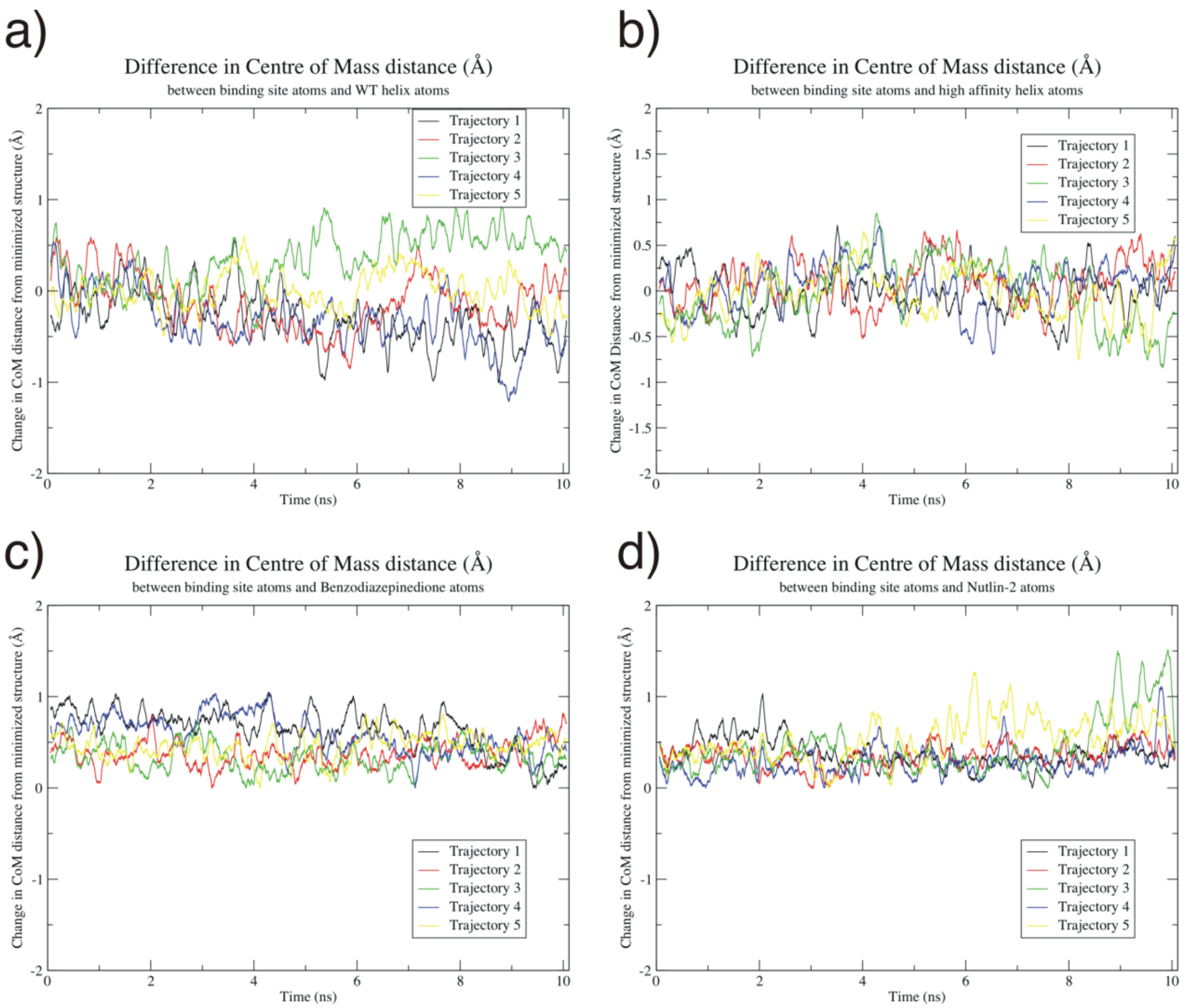

Figure S8: Difference in center of mass distance (in Å) between hDM2 and its binding partner from initial minimized structure, for a) wild-type helix; b) high-affinity helix; c) benzodiazepinedione; and d) Nutlin-2.

Center of mass distance between protein and ligand: The center of mass distance between *h*DM2 and the ligands remains close to their starting values, showing that all ligands are tightly bound in the *h*DM2 binding pocket. Monitoring this distance allows us to determine changes in the location in the pocket of the binding partner. In the case of simulations starting from known structures this value should remain close to zero since the system is likely to already be at a minima. Figure S8 shows the deviations in distance between the center of mass of the *h*DM2 protein and the center of mass of the

ligand binding partner. In Figure S8a we see that the wild-type helix distance varies between -1 Å and +1 Å and may have the most variability between each of the replicates. This may indicate that the native p53 helix is quite flexible in the binding site and that it may be possible to identify more tightly binding partners. The high-affinity helix in Figure S8b shows less variability with minima and maxima closer to -0.5 Å and +0.5 Å. The benzodiazepinedione and Nutlin-2 families of simulations in Figure S8c and S8d, respectively, appear to slightly increase center of mass distance over time. While the two peptide simulations in S8a and S8b oscillate around a zero value, the two small-molecule simulations in S8c and S8d oscillate around mean values of approximately +0.2 Å, which is within the error range of the simulation. These values are nevertheless also very stable.

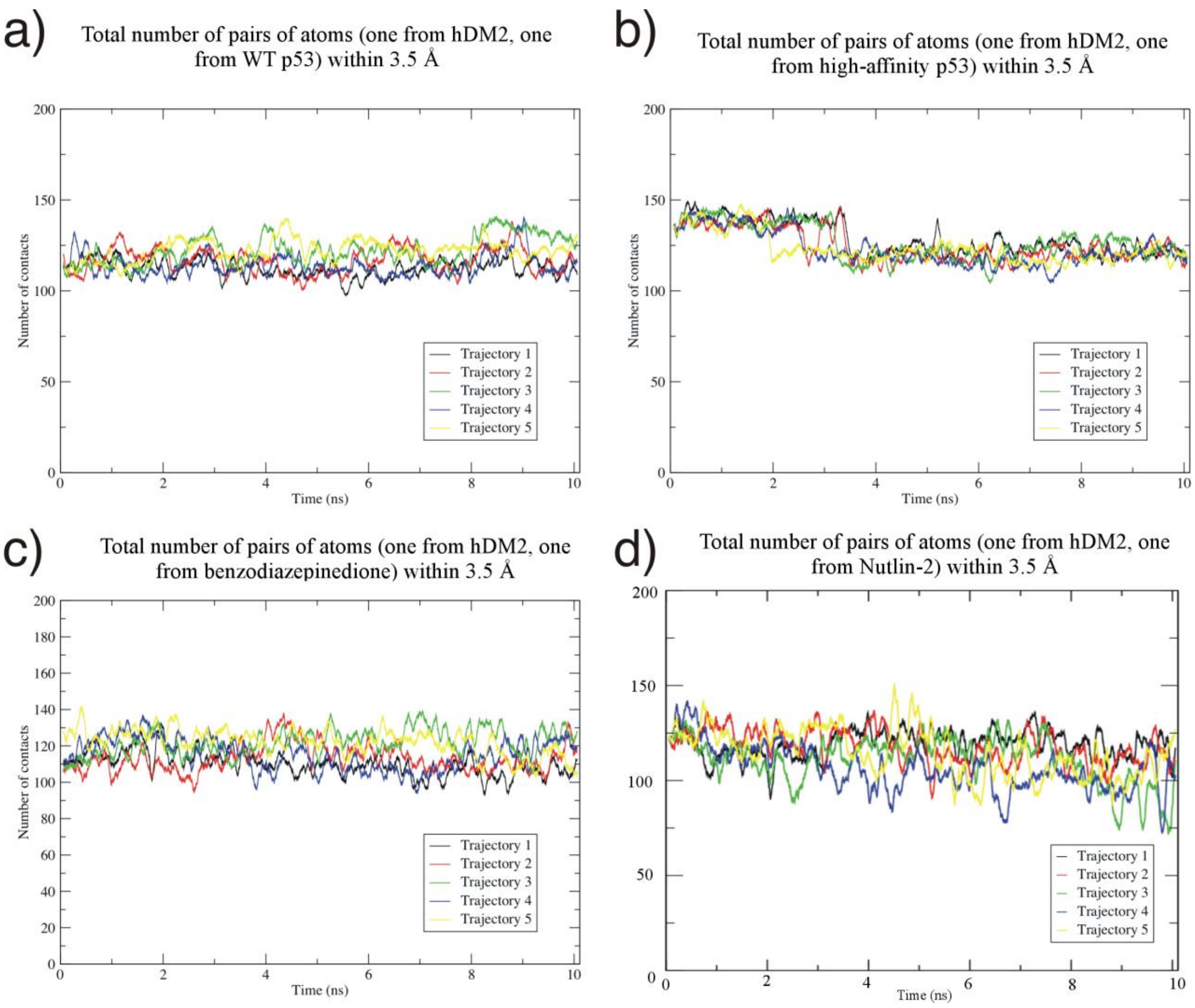

Figure S9: Total number of contacts, pairs of atoms that are within 3.5 Å of each other, one from hDM2 and one from complex structure: a) wild-type helix; b) high-affinity helix; c) benzodiazepinedione; and d) Nutlin-2. Average number of contacts does not change significantly over time in any complex, indicating stable bound conformations.

Total number of protein-ligand contacts: the number of intermolecular pairs of atoms between hDM2 and the ligand that are within a 3.5 Å threshold remain constant during the simulations, implying that the complexes are already in a stable binding pose. We also monitored the total number of protein-ligand contacts (Figure S9), where a contact is defined as a pair of atoms, one from *h*DM2 and one from the ligand, located within 3.5 Å of each other. Since the van der Waals radius of a carbon atom is approximately 1.7 Å, this means that we are essentially counting the number of contacts of the

complexed molecule with the *h*DM2 protein. This gives us a very rough measure of how tightly bound the ligand is. In the case of co-crystallized ligands, we might expect the number of contacts to stay roughly constant for the duration of the simulations, since it should already be in a global minimum. In the high-affinity helix simulations in Figure S9b, all simulations start with a larger number of contacts than they finish with, moving from approximately 140 contacts to approximately 110 contacts. All simulations make this transition between 2 ns and 4 ns of simulation time. Once the high-affinity helix simulation settles to its new number of contacts, this value is roughly the same as that of the wild-type helix. This is unsurprising because while the wild-type helix is slightly longer than the high-affinity helix, both helices share the same contact region. Much of the increase in affinity of the high-affinity helix appears to be from decreasing the overall helix length to the minimum amount required to maintain helicity, while substituting residues on the solvent exposed face of the peptide for those that are helix stabilizers with a high helix propensity and additionally have hydrophillic properties.[7] Total number of contacts for both benzodiazepinedione and Nutlin-2 (Figure S9c and S9d respectively), remain constant with values around 110, even though one might expect to observe a larger number of contacts for a ligand with a larger number of atoms. We see that in the case of Nutlin-2 and benzodiazepinedione the number of contacts is directly comparable to both of the p53 helices.

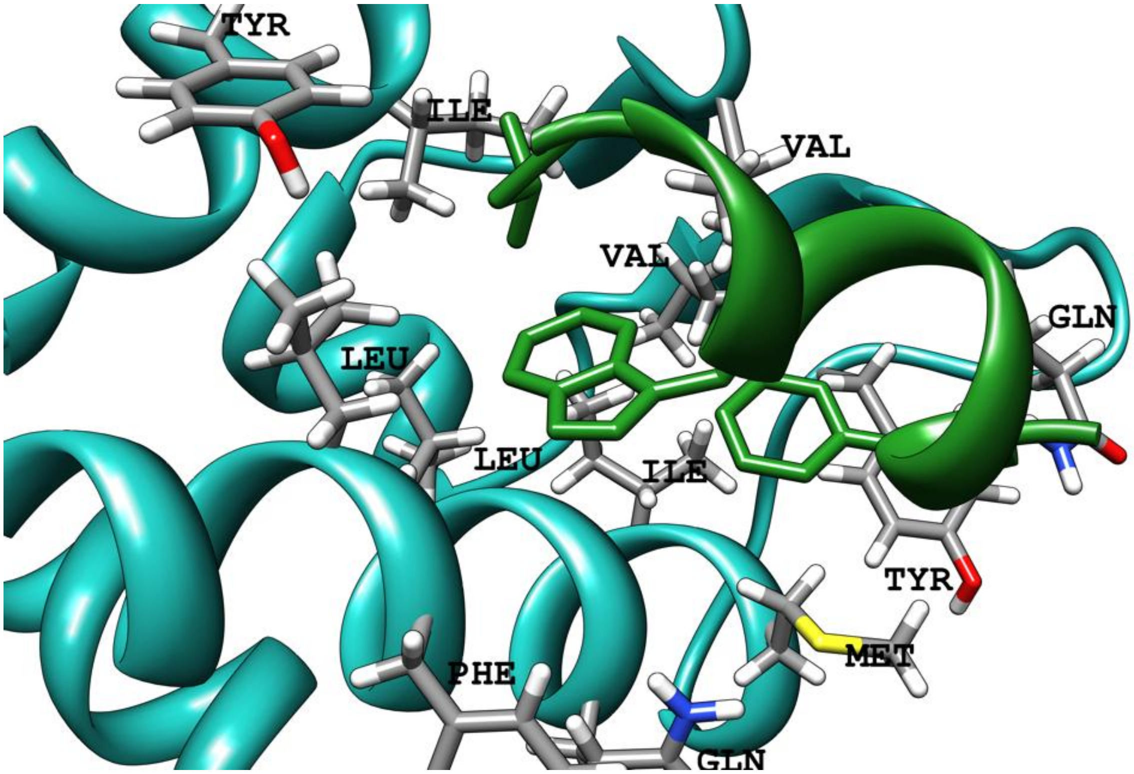

Figure S10: Binding site residues that are investigated in dihedral angle sampling analysis shown in table 1. hDM2 protein backbone shown in ribbon style(cyan); high-affinity p53 helix (green); residues (atom colours).

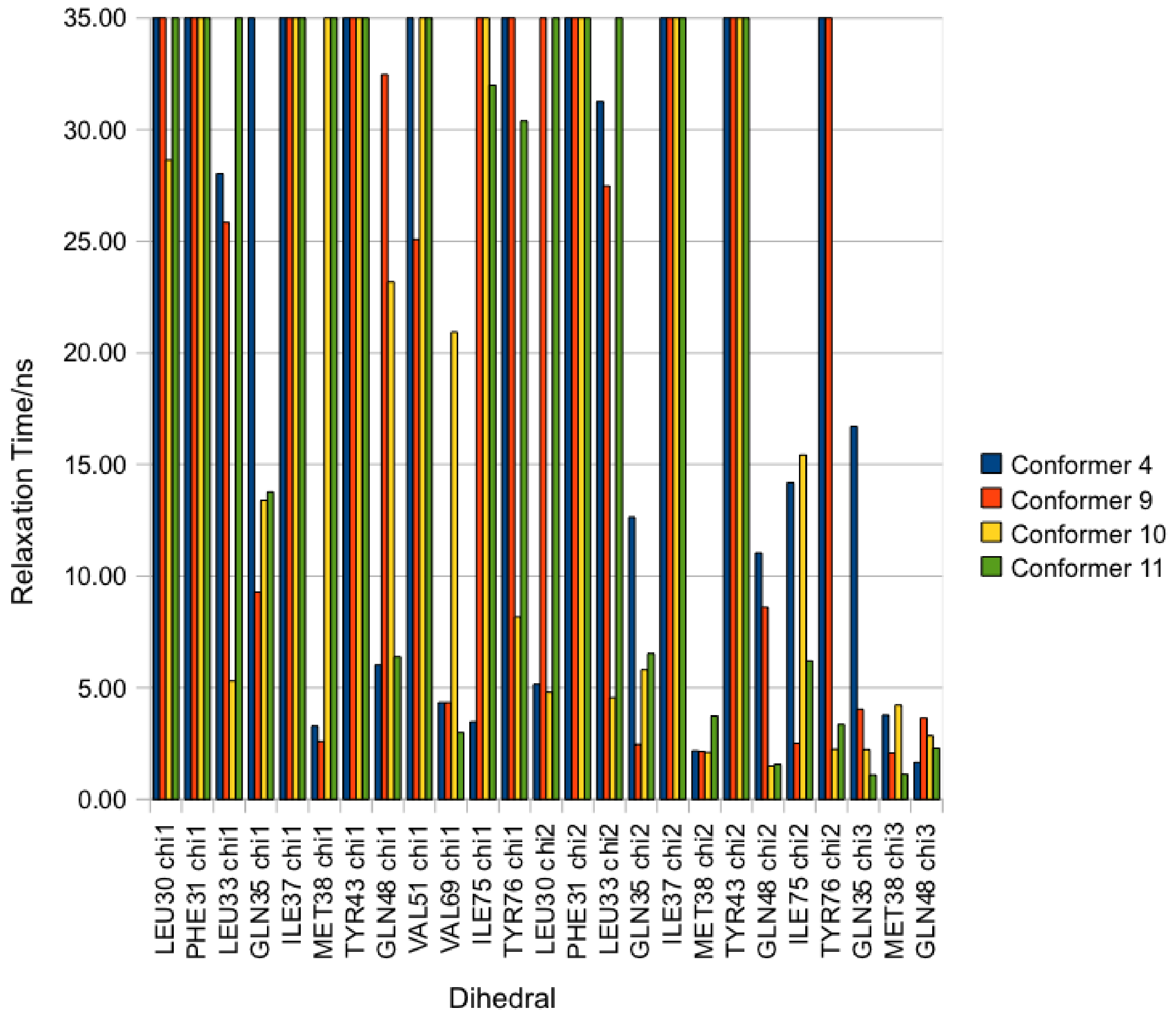

Figure S11: Estimated relaxation times for dihedral angles from the hDM2 binding site for parallel conformations of bound arylamide compounds as calculated by fitting a function of the form y=exp(-x/a) to the autocorrelation function for the dihedral angle.

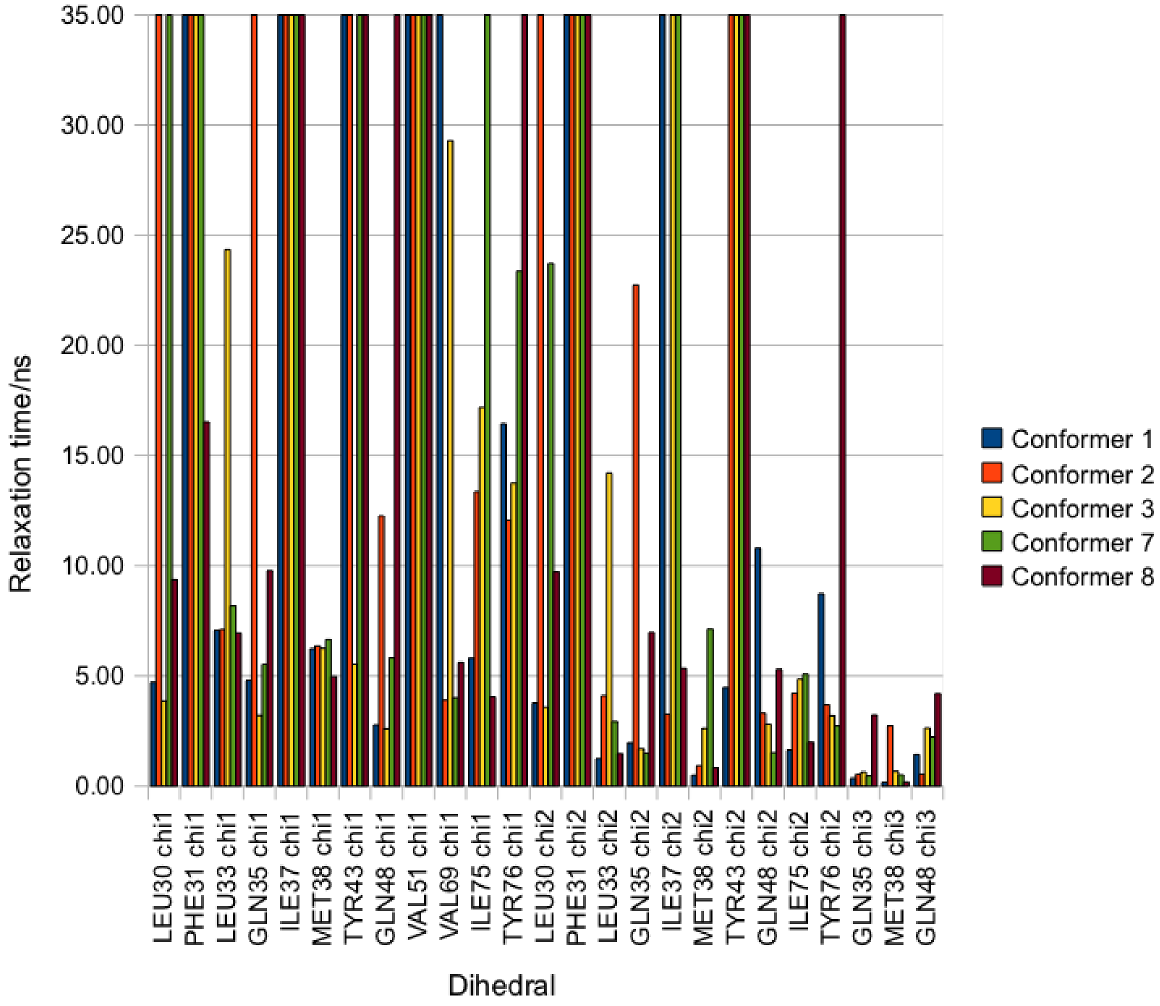

Figure S12: Estimated relaxation times for dihedral angles from the hDM2 binding site for anti-parallel conformations of bound arylamide compounds as calculated by fitting a function of the form y=exp(-x/a) to the autocorrelation function for the dihedral angle.

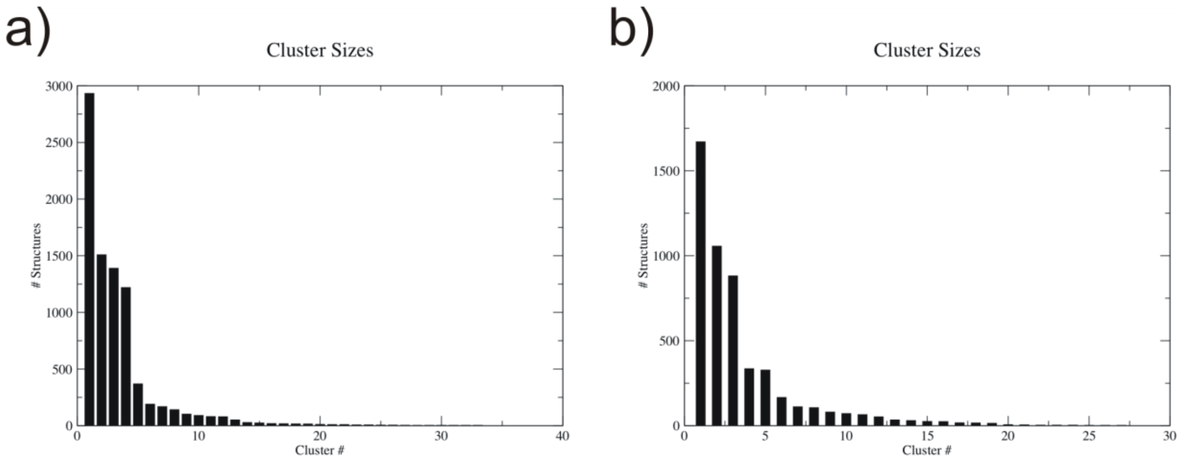

Figure S13: Number of conformers fitting clusters defined at an RMS threshold (of 1.5 Å) from the final 17 ns of simulation, sampled every 10 ps for a) 5 anti-parallel simulations and b) 3 parallel simulations.

Charge derivation

Charge calculations

Charge calculations were performed to determine which method for charge calculation would be most appropriate for the *h*DM2-arylamide system. We compared AM1 BCC semi-empirical calculations to Hartree-Fock calculations using the HF 6-31G* basis set.

Generating conformers for AM1 BCC calculations

Conformers were generated for AM1 BCC calculations using OpenEye OMEGA. Parameters were selected based on those most likely to produce ligand conformations that are bioactive. Therefore, an energy window of 25 kcal mol^{-1} was used,[8] and RMS tolerance and maximum number of generated conformers was set so as to generate a wide range of 350 conformers. This meant a value between 0.45 Å and 0.55 Å was used for the RMS cutoff and maxconfgen was set to 10,000. This resulted in: 310 Phe-Trp-^{i}Pr; 361 Phe-Nap-^{i}Pr; 380 Val-Phe-Pr; and 361 CH_3-CH_3-CH_3 conformations.

AM1 BCC calculations

Semi-empirical AM1 BCC charge calculations were performed for each of the conformers generated, using OMEGA (as described in the above section) with the Antechamber program supplied by AMBER 8.[9] Calculation of charge for each conformer took on the order of several minutes. The mean and variance for the charge of each atom were then calculated using a custom script for the R statistical computing language.[10]

Quantum calculations

Quantum calculations were performed using the Hartree-Fock level of theory and the HF 6-31G* basis set. Initial conformations of compounds were generated in Gaussview. The REDIII.1 software was used in tandem with Gaussian 03 to perform the calculations, which consisted of a geometry optimization followed by Molecular Electrostatic Potential calculation and charge fitting using the RESP method.[11,12] This scheme was chosen since it most closely resembles the method that was originally employed for deriving charge parameters for the AMBER force field. Calculations were

performed on full arylamide compounds with the Phe-Trp-Leu substitution pattern, and the -CH_3-CH_3-CH_3 substitution pattern. Full molecule calculations took on the order of one week to complete when carried out using a 2.2 GHz Opteron processor. Fragment compounds containing the central benzene ring, carboxylic acid, primary amine and a single substitution of CH_3 or Trp were also investigated. These calculations took approximately 2 days to complete using a single processor.

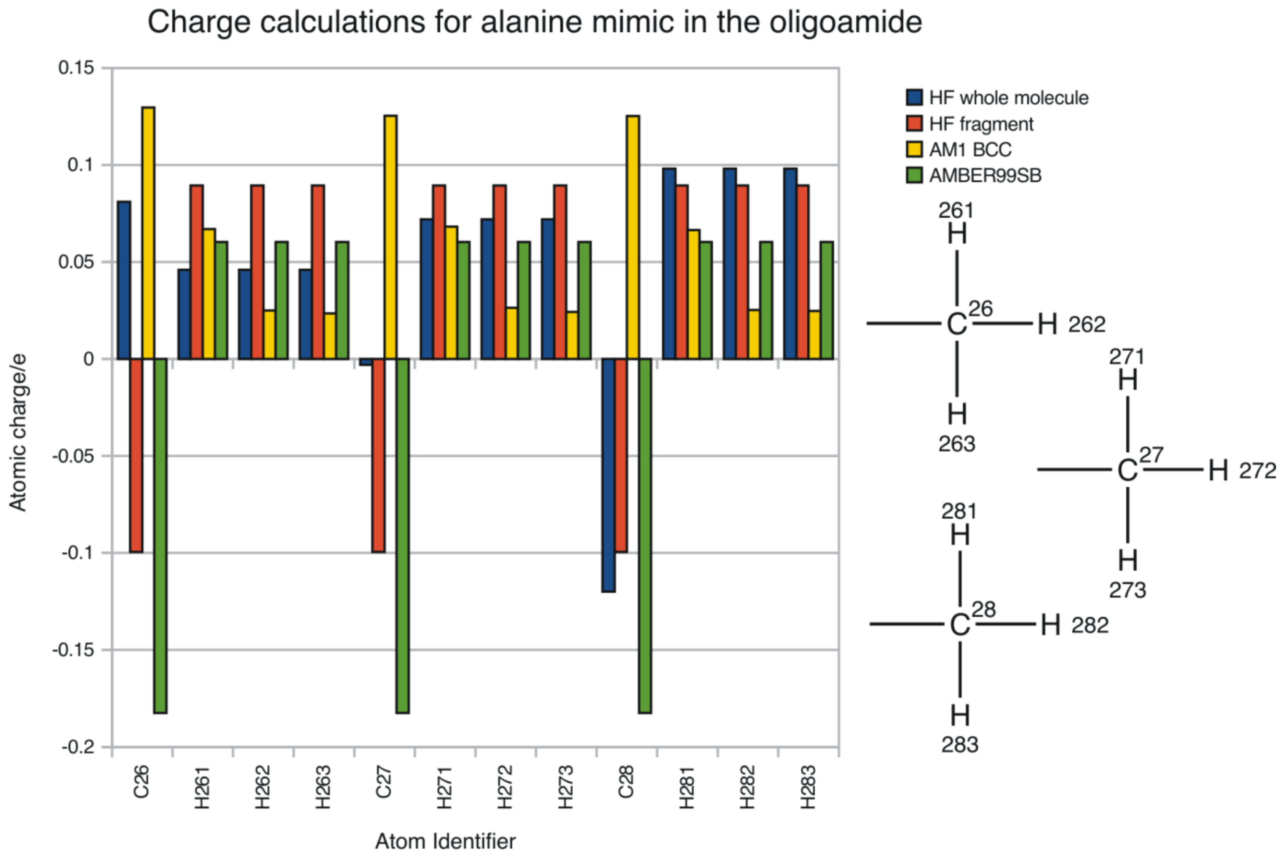

Figure S14: Comparison of charge calculation methods applied to the triple alanine substituted arylamide. Results for the HF 6-31G* level of theory applied to a full molecule (blue); HF 6-31G* level of theory applied to a fragment containing the alanine side-chain (orange); the mean value from 361 conformations of the full compound using the AM1 BCC level of theory (yellow); and the charge values specified for the corresponding alanine side-chain atoms in the AMBER99sb force field (green).

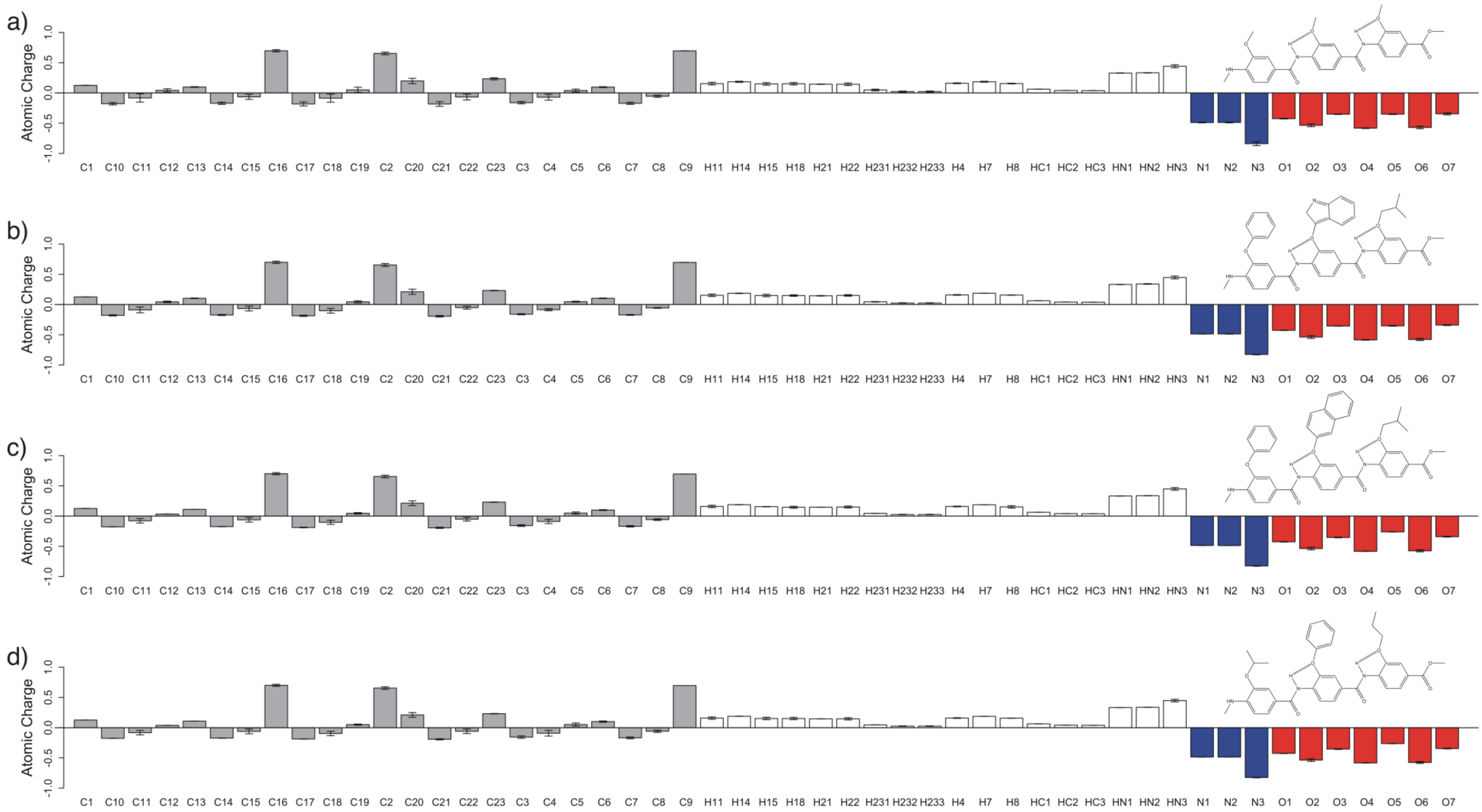

Figure S15: Atomic charge calculated using the AM1 BCC charge method implemented in the Antechamber program from AmberTools 1.2. Mean and 95 % confidence interval was calculated for the stated number of conformations generated using the OMEGA package provided by OpenEye software. a) 361 structures from a triple -CH_3 substituted compound; b) 310 structures from a Phe-Trp-^{i}Pr mimic; c) 361 structures from a Phe-Nap-^{i}Pr mimic; d) 380 structures from a Val-Phe-Pr compound.

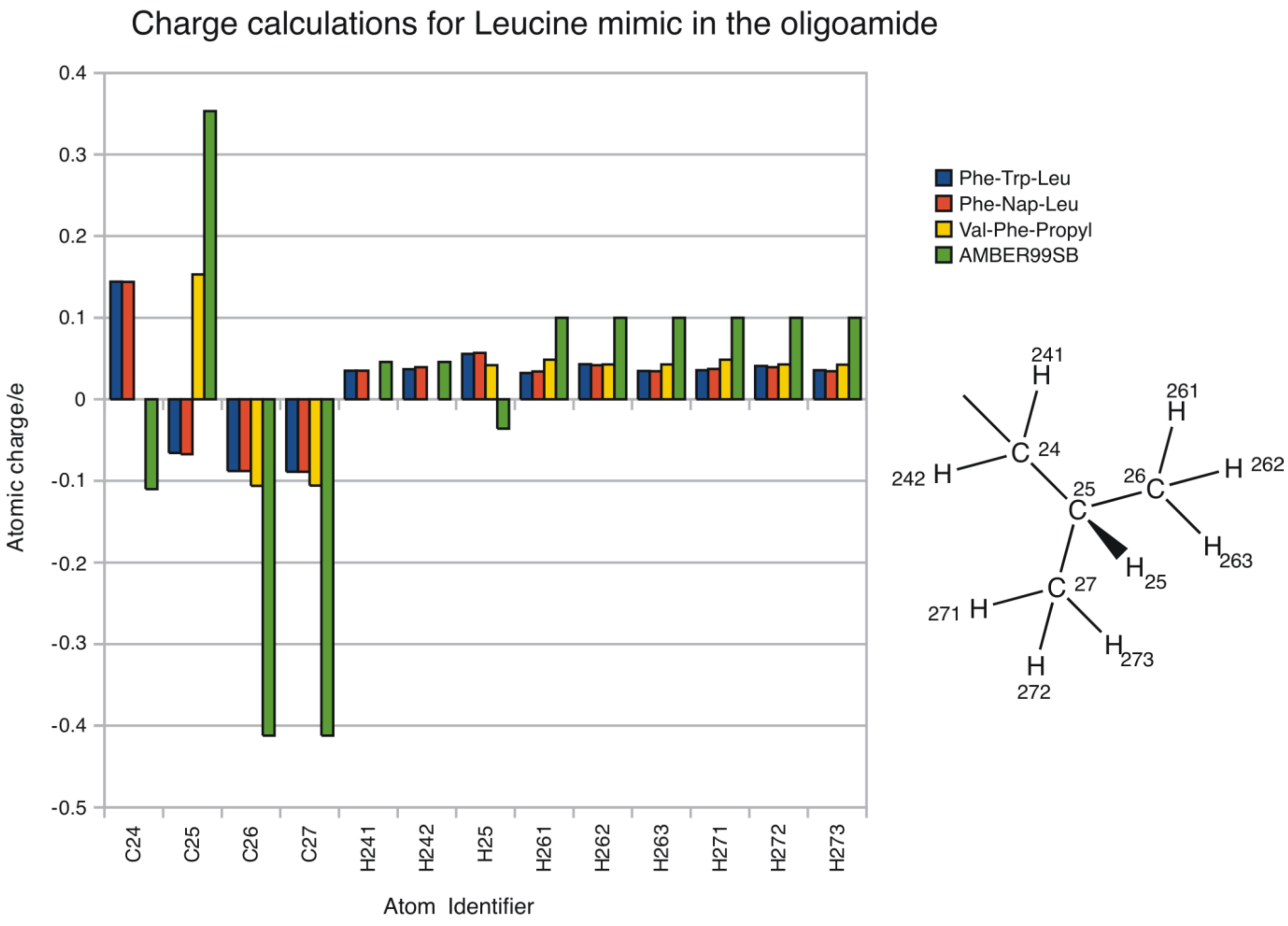

Figure S16: AM1 BCC charge calculations for Leucine side-chain mimics. Mean values calculated from the result of: 310 conformations from a Phe-Trp-^{i}Pr compound (blue); 361 conformations from a Phe-Nap-^{i}Pr compound (orange); 380 conformations from a Val-Phe-Pr compound (yellow); and AMBER99sb Leucine side-chain charges for comparison (green).

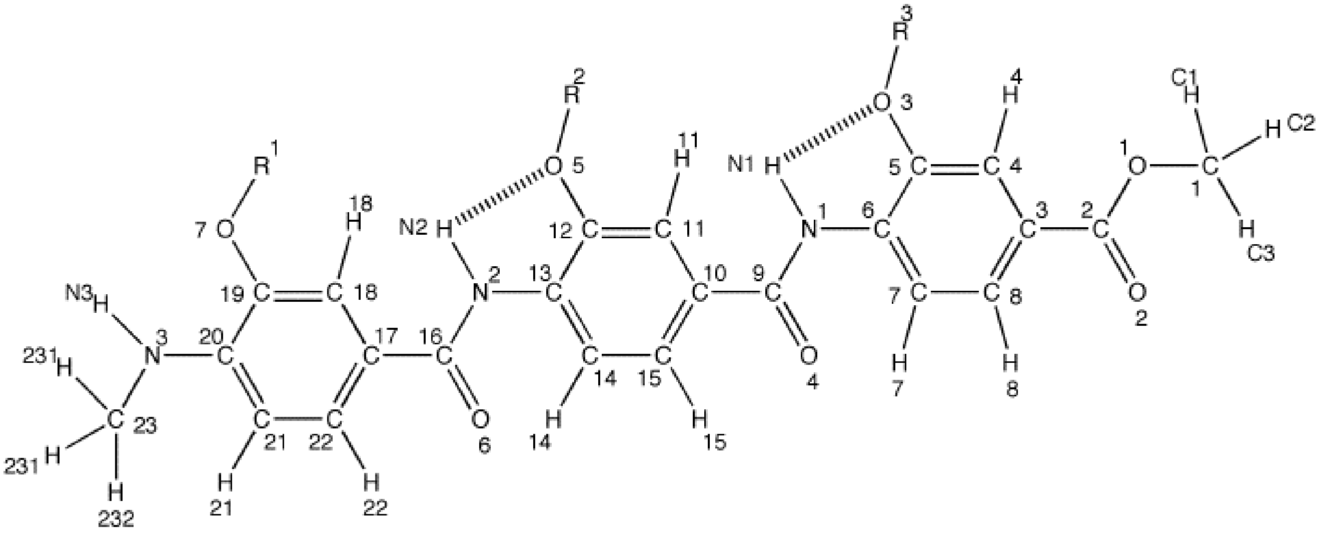

Figure S17: Schematic of the atom labelling scheme used in the charge calculation work for backbone atom labelling, showing the atomic element and the number used to identify specific atoms.

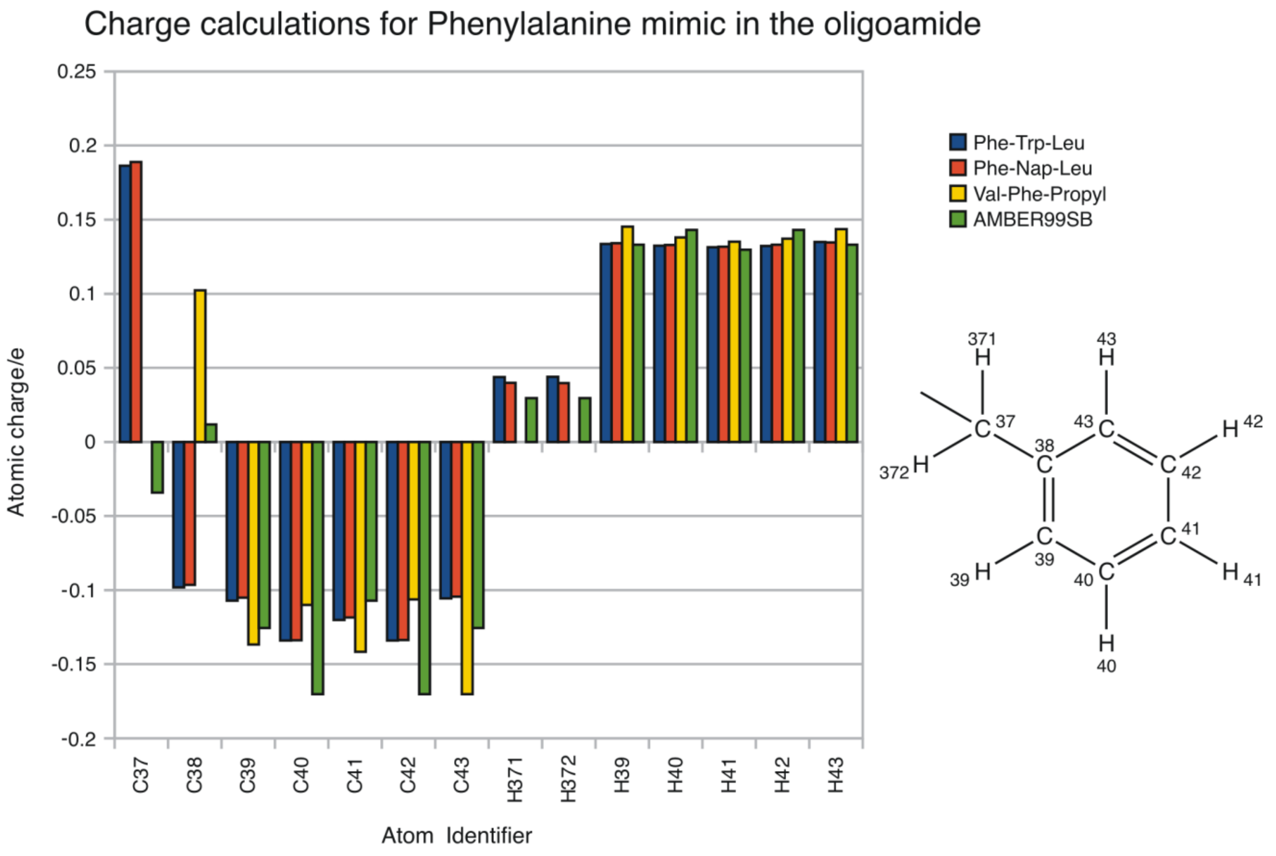

Figure S18: AM1 BCC charge calculations for Phenylalanine side-chain mimics. Mean values calculated from the result of: 310 conformations from a Phe-Trp-^{i}Pr compound (blue); 361 conformations from a Phe-Nap-^{i}Pr compound (orange); 380 conformations from a Val-Phe-Pr compound (yellow); and AMBER99sb Leucine side-chain charges for comparison (green).

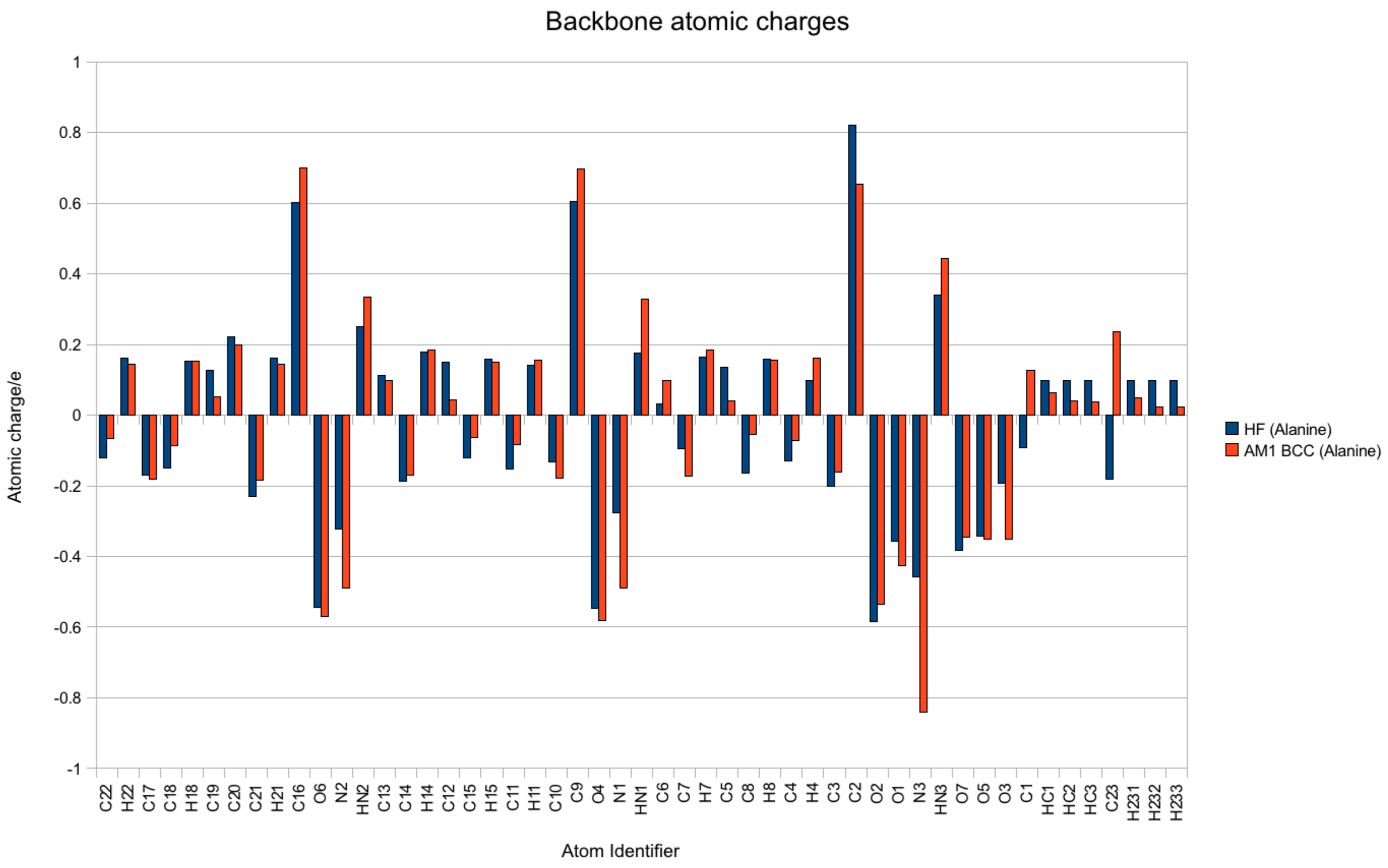

Figure S19: Backbone atomic charges calculated using the HF6-31G* level of theory (blue) compared to backbone atomic charges calculated using the AM1 BCC level of theory (orange). Full QM calculations were carried out using Gaussian and the REDIII.1 software package; semi-empirical QM calculations were carried out using Antechamber from AmberTools 1.2. Full details are given in the Methods section of the main paper.

Arylamide charge calculations

Two charge calculation methods were evaluated: the AM1 BCC semi-empirical method implemented with Antechamber from the AMBER package and the high level Hartree-Fock molecular electrostatic potential calculation method followed by RESP charge fitting using the REDIII.1 program.[9,11,13] Both methods calculated backbone charges for the compound shown in Figure S17. Additionally side-chain charges for positions R_1, R_2 and R_3 were calculated for the four compounds shown in 2D to the right of Figure S15, for comparison these values were compared to related side-chain charges from the AMBER99 force fields. The two charge calculation methods were chosen since they are consistent with the charge calculation methods used to calculate charges for the AMBER force fields. In particular, Hartree-Fock calculations using the 6-31G* basis set followed by RESP charge fitting was used to derive charge parameters for the original AMBER94 force field described by Cornell *et al*.[14] The AM1 BCC charge calculation is considered as an alternative to the HF 6-31G* method, since it is several orders of magnitude faster, while also being parameterized such that it should reproduce charges calculated using the HF 6-31G* basis set and the RESP method.[13] Comparison is made between backbone AM1 BCC charges for arylamide compounds calculated for a large number of conformers of four compounds described in Figure S15. Side-chain charges for Leucine and Phenylalanine mimics were also compared using the AM1 BCC charge method. We then compared two HF 6-31G* methods for backbone charge calculation. In one, we calculated for the entire arylamide compound shown in Figure S17. In the second, we split the compound into one of three fragments that when combined could describe the entire molecule. Additionally we compared to the AM1 BCC method. The fragment method for calculating HF 6-31G* method allows for increased speed of computation. For example, if we wanted to simulate a selection of arylamide compounds derived from a library of three side-chains, we would need to perform 27 full molecule arylamide simulations, compared to 3 individually less expensive fragment HF 6-31G* calculations. The HF 6-31G* full molecule and fragment calculations were finally compared to the AM1 BCC charge method for side-chain mimics of alanine and

tryptophan, which were in turn compared to the values provided with the AMBER force fields.

Arylamide Backbone charges

The first comparison made was between backbone charges calculated using the AM1 BCC charge method. Figure S15 shows backbone calculations for a) -CH_3-CH_3-CH_3; b) Phe-Trp-^{i}Pr; c) Phe-Nap-^{i}Pr; and d) Val-Phe-Pr. It is clear that there is not much variation between each of the backbones, indicating that the method provides a reasonable consensus. This conclusion is supported by very small calculated error bars. Of note are C16, C2 and C9, which comprise the 3 carbons that form the amide bond between aryl groups that have the largest positive charges for carbon atoms: +0.699 e, +0.655 e, and +0.697 e. We also note that since the arylamide compound is an oligomer, we see a degree of symmetry in the results. That is, C20, C13 and C6 (carbon atoms that exist at equivalent positions in the oligomer) are all slightly positive with charges of +0.199 e, +0.098 e, and +0.097 e, respectively. Nitrogens N1 and N2 carry almost identical negative charges, while nitrogen N3 carries a larger negative charge. We might expect more variation in the charges of the ether oxygens O3, O5 and O7 that occupy positions that mimic positions of the Cα at positions i, i+3 and i+7 on a superposed helix. There is little variation in the charge calculated for these oxygens; however, they are less negative than the oxygen atoms that form the amide bonds.

Arylamide side-chain charges

In Figure S16 we look at how the AM1 BCC charge calculation varies for side-chains that attempt to mimic Leucine/Valine side-chains due to the compound to which it is attached. In all cases the backbone is the same. However, in the case of the Val-Phe-Pr compound, the side-chain of interest is at the N-terminus of the compound rather than the C-terminus of the compound as in the Phe-Trp-^{i}Pr and Phe-Nap-^{i}Pr compounds. These compounds are shown in 2D to the right of figures S15d, S15b and S15c respectively. Bars for C24, H241 and H242 are missing for the Val-Phe-Pr compound since these atoms are not present in this compound. The strong electronegativity of the ether oxygen is clear from

the large positive charge for C24 in Phe-Trp-Leu and Phe-Nap-Leu and for C25 in the case of Val-Phe-Propyl. The two carbons farthest from the attachment ether, C26 and C27 have similar positive charge values in all three compounds. As a comparison, charge values for the Leucine side-chain contained in the AMBER99 force field are included. They follow the pattern C24 slightly negative, C25 large positive, C26 and C27 negative, with values: -0.110 e, +0.353 e, -0.412 e, and -0.412 e respectively. Hydrogen values in all cases are much more comparable between all data sets. AMBER99sb hydrogens attached to C26 and C27 are approximately double those calculated with the AM1 BCC charge methods.

Figure S18 provides a similar comparison to the above Leucine side-chains for Phenylalanine mimics from the Phe-Trp-Leu, Phe-Nap-Leu and Val-Phe-Propyl compounds. The Phenylalanine mimic in the Val-Phe-Propyl compound is missing the alkyl carbon C37 and corresponding hydrogens H371 and H372, as can be seen to the right of Figure S15d. As a result, carbon C37 bound to the ether oxygen has a charge of about +0.1 e compared to ~ +0.2 e for C37 and -0.1 e for C38 from Phe-Trp-Leu and Phe-Nap-Leu compounds that contain the alkyl carbon before the benzene ring. Despite this difference the charge on the other ring atoms is generally comparable between all three compounds. Phenylalanine atoms, with the exception of C37 and C38 (and corresponding hydrogen atoms), also have comparable charges in the AMBER99 force field.

AM1 BCC charge calculations as implemented here do not take into account symmetry of atoms in a calculation. For example, in Figure S9 the phenylalanine side-chain calculations for C39 and C43 in the Va-Phe-Propyl compound have charge values of -0.137 e and -0.170 e, respectively. Yet they are indistinguishable particles. Thus, any model should ideally treat them as identical, meaning that they should end up with the same charge.

It is reassuring that the conformation of the molecule that OMEGA generates does not have a large effect on the charge of the molecule as shown by the small error bars observed in Figure S15. Additionally the calculations appear to arrive at a consensus value for the charge of the backbone that is

independent of the substitution of pattern for the side-chain groups. It should be noted that the side-chain mimics investigated in this study are all alkyl or aryl groups with no net charge, so some care must be taken if these compounds are investigated.

Full quantum mechanical vs. semi-empirical charge calculations

We next look at the results for HF 6-31G* backbone charge calculations compared to AM1 BCC charge calculations for R_1=CH_3,R_2=CH_3,R_3=CH_3 substituted compounds. The results are presented in Figure S19 and generally show broad agreement. The main disagreement is for C23, which is part of the N-terminal methyl cap. This capping group is not present in the final arylamide simulations presented paper, as it is not necessary for performing accurate free energy calculations. Thus disagreement between the two charge methods for this atom have no impact on the free energy calculations described later. Since the two charge methods agree well in the case of the backbone calculations, we must make a final comparison of charge calculation methods for side-chains before we can decide which charge method to proceed further with.

In Figure S14 we compare the two HF 6-31G* charge methods with AM1 BCC and provide comparison to AMBER charges for Alanine. Since we calculated the value for the CH_3 side-chain only once using the fragment HF 6-31G* method, we include the results against each side-chain that was calculated using the HF 6-31G* and AM1 BCC methods. We might expect that the full molecule methods for side-chain charge calculation should produce the same value for each $CH_{3,}$ thus validating the fragment HF 6-31G* method as a suitable candidate. However, we see that it disagrees with the full molecule side-chain calculations for the carbons comprising the two side-chains closest to the C-terminal. Full molecule calculations in this case yield values of +0.081 e and -0.003 e compared to -0.100 e for the fragment calculation. However, the fragment and full-molecule calculations are in strong agreement with the value for the N-terminal side-chain carbon atom, -0.100 e and -0.120 e, respectively. The calculations for the hydrogens in the CH_3 side-chains once again show the problem

that the AM1 BCC method has with assigning differing charges to indistinguishable atoms that is not visible in either of the HF 6-31G* methods. The CH_3 side-chain calculations are the first point where we see disagreement between the HF 6-31G* and AM1 BCC charge methods. The AM1 BCC charge method produces very similar values for all CH_3 side-chains, whilst the HF 6-31G* method produces values that decrease from positive at the C-terminal substituent to negative at the N-terminal substituent. This in turn encourages the bound hydrogens to increase their charge values from approximately +0.05 e to +0.1 e from C- to N-terminal. In this case, the HF 6-31G* method appears to take into account the dipole moment of the molecule, since the C-terminus carries a negative charge and thus is likely to have an electron donating character, whilst the N-terminus carries a positive charge and thus is likely to have an electron withdrawing characteristic. Finally, we note that the AMBER charges most closely resemble those of the fragment CH_3 compound with the HF 6-31G* method. This is in many ways expected since the fragment method essentially creates a compound that is most similar to those created when the original AMBER force field was parameterized.

Tryptophan side-chain charge calculations are also performed using AM1 BCC and fragment HF 6-31G* methods. Full molecule HF 6-31G* calculations are not evaluated since these calculations did not converge to an answer for over a week of CPU time. We see broad agreement with the charges calculated for atoms from both methods and the AMBER charges. There are some notable exceptions, the first is the AM1 BCC charge of +0.2 e for C28, the alkyl carbon bound to the backbone ether oxygen, compared to charges much closer to 0 e in the case of the HF 6-31G* calculation and those provided with the AMBER force field. We also note a value close to -0.2 e for the AM1 BCC charge assigned to N4 (the tryptophan nitrogen), compared to a value slightly over -0.4 e for the HF 6-31G* calculation.

Charge calculations summary

In general, we have shown that the two charge calculation methods appear in broad agreement in the contexts to which they have been applied. It is likely that the HF 6-31G* method would provide more robust results, although the increased calculation time required is a very considerable additional overhead. If this type of charge calculation is to be used in an extensive modelling study, it is likely that the AM1 BCC charge calculations should be sufficient and necessary in order to generate models in sufficient time. HF 6-31G* methods, or perhaps some intermediate level of theory could be used, as in the study by Vemparala *et al.*[15] These calculations are required to generate ab-initio torsional parameters for some of the compounds. It would then be possible to use these calculations and RED to do RESP charge fitting.

In addition to investigating the variation in charge parameters we can ask how much incorrectly assigning charge parameters to our molecules might affect the results of a free energy calculation. The GAFF force field was used to determine the bonded parameters, whilst a variety of MP2, B3LYP, HF 6-31G*, AM1 CM2 and AM1 BCC charge calculations were performed. The results from these alchemical free energy calculations were then compared to experiment. In this study, all computed errors for free energies of hydration were less than 0.1 kcal mol^{-1} which is less than the reported 0.2 kcal mol^{-1} error reported in the study from which the experimental data is taken.[16] This report provides extra weight to support a policy of spending some time to ensure that the calculated charge values are as accurate as possible, whilst bearing in mind that the additional speed provided by AM1 BCC methods probably outweighs any disadvantages due to inaccuracies in these charge calculations. In addition, it can be noted that AM1 BCC charge methods have been used in other studies of hydration free energies to good effect and have also been used in successful calculations of protein-ligand free energy of association.[17,18]

References

(1) Brakoulias, A.; Jackson, R. M. *Proteins* **2004**, *56*, 250-60.

(2) Rocchia, W.; Alexov, E.; Honig, B. *J. Phys. Chem. B* **2001**, *105*, 6754-6754.

(3) Pettersen, E. F.; Goddard, T. D.; Huang, C. C.; Couch, G. S.; Greenblatt, D. M.; Meng, E. C.; Ferrin, T. E. *J. Comput. Chem.* **2004**, *25*, 1605-12.

(4) Kyte, J.; Doolittle, R. F. *J. Mol. Biol.* **1982**, *157*, 105-32.

(5) Hernandes, M. Z.; Cavalcanti, S. M. T.; Moreira, D. R. M.; Azevedo Junior, W. F. de; Leite, A. C. L. *Curr. Drug Targets* **2010**, *11*, 303-14.

(6) Willis, B. T. M.; Pryor, A. W. *Thermal vibrations in crystallography*; Cambridge University Press: Cambridge, 1975; p. 296.

(7) Pace, C. N.; Scholtz, J. M. *Biophys. J.* **1998**, *75*, 422-7.

(8) Kirchmair, J.; Wolber, G.; Laggner, C.; Langer, T. *J. Chem. Inf. Model.* **2006**, *46*, 1848-61.

(9) D.A. Case, T.A. Darden, T.E. Cheatham, III, C.L. Simmerling, J. Wang, R.E. Duke, R.; Luo, K.M. Merz, B. Wang, D.A. Pearlman, M. Crowley, S. Brozell, V. Tsui, H. Gohlke, J.; Mongan, V. Hornak, G. Cui, P. Beroza, C. Schafmeister, J.W. Caldwell, W.S. Ross, A.; Kollman, P. A. AMBER **2004**.

(10) R: A Language and Environment for Statistical Computing **2009**.

(11) E. Vanquelef; Simon, S.; Marquant, G.; Garcia, E.; Klimerak, G.; Delepine, J. .; Cieplak, P.; Dupradeau, . F.-Y. R.E.D. Server **2010**.

(12) M. J. Frisch, G. W. Trucks, H. B. Schlegel, G. E. Scuseria, M. A. Robb, J. R. Cheeseman, J. A. Montgomery, Jr., T. Vreven, K. N. Kudin, J. C. Burant, J. M. Millam, S. S. Iyengar, J. Tomasi, V. Barone, B. Mennucci, M. Cossi, G. Scalmani, N. Rega, G. A. Pet, and J. A. P. Gaussian 03 **2004**.

(13) Jakalian, A.; Jack, D. B.; Bayly, C. I. *J. Comput. Chem.* **2002**, *23*, 1623-41.

(14) Cornell, W. D.; Cieplak, P.; Bayly, C. I.; Gould, I. R.; Merz, K. M.; Ferguson, D. M.; Spellmeyer, D. C.; Fox, T.; Caldwell, J. W.; Kollman, P. A. *J. Am. Chem. Soc.* **1995**, *117*, 5179-5197.

(15) Vemparala, S.; Ivanov, I.; Pophristic, V.; Spiegel, K.; Klein, M. L. *J. Comput. Chem.* **2006**, *27*, 693-700.

(16) Mobley, D. L.; Chodera, J. D.; Dill, K. A. *J. Chem. Phys.* **2006**, *125,* 084902.

(17) Mobley, D. L.; Dumont, E.; Chodera, J. D.; Dill, K. A. *J. Phys. Chem. B* **2007**, *111*, 2242-54.

(18) Mobley, D. L.; Graves, A. P.; Chodera, J. D.; McReynolds, A. C.; Shoichet, B. K.; Dill, K. A. *J. Mol. Biol.* **2007**, *371*, 1118-34.
